# Sex Differences in Amyloid Pathology by Race, Ancestry, and Apolipoprotein E ε4 in an Admixed Autopsy Sample

**DOI:** 10.1001/jamaneurol.2026.0054

**Published:** 2026-02-23

**Authors:** Maison Abu Raya, Claudia Kimie Suemoto, Vitor Ribeiro Paes, Renata Elaine Paraizo Leite, Carlos Augusto Pasqualucci, Michel Satya Naslavsky, Roberta Diehl Rodriguez, Ricardo Nitrini, Eduardo Ferriolli, Isabel Elaine Allen, Renaud La Joie, Lea T. Grinberg

**Affiliations:** 1 Global Brain Health Institute, University of California, San Francisco; 2 Memory and Aging Center, University of California, San Francisco; 3 Division of Geriatrics, Department of Internal Medicine, University of São Paulo Medical School, São Paulo, Brazil; 4 Physiopathology in Aging Laboratory, Department of Pathology, University of Sao Paulo Medical School, Sao Paulo, Brazil; 5 Department of Genetics and Evolutionary Biology, Institute of Biosciences, University of São Paulo, Sao Paulo, Brazil; 6 Department of Neurology, University of São Paulo Medical School, Sao Paulo, Brazil; 7 Laboratory Medicine and Pathology Department, Mayo Clinic, Jacksonville, Florida

## Abstract

**Importance:**

Sex and racial or ancestral disparities in Alzheimer disease remain incompletely understood; autopsy studies that examine amyloid, tau, and genetic factors are scarce.

**Objective:**

To test whether neuritic plaque burden and cognitive outcomes differ by sex and whether sex modifies the effects of apolipoprotein E ε4 (*APOEε4*), informant-reported race, and African ancestry.

**Design, Setting, and Participants:**

This was a cross-sectional study using postmortem neuropathological data from the Biobank for Aging Studies, University of São Paulo, São Paulo, Brazil. A total of 2268 autopsies from a population-based, diverse clinicopathological sample were collected between April 2004 and March 2025.

**Exposures:**

Sex, informant-reported race (Black, White), African ancestry proportion, and *APOEε4* carrier status.

**Main Outcomes and Measures:**

Neuritic plaque burden (Consortium to Establish a Registry for Alzheimer’s Disease [CERAD] score), and cognitive function (Clinical Dementia Rating–Sum of Boxes [CDR-SB]). Ordinal logistic regression examined association of sex with CERAD scores and 2- and 3-way interactions among sex, race, ancestry, and *APOEε4*; adjusting for age, education, vascular factors, and Braak stages. Linear models related pathology to CDR-SB, adding copathologies.

**Results:**

The analysis included 2268 autopsies (median [IQR] age, 74.8 [63.8-83.3] years; 1152 [51% male] and 1116 [49%] female; 802 [35%] Black and 1466 [65%] White; other race groups not included owing to small numbers); female individuals were older than male individuals and more likely to exhibit cognitive impairment (CDR global score ≥0.5). Female individuals had higher plaque burden than male individuals (unadjusted odds ratio [OR], 1.97; 95% CI, 1.67-2.29; *P* < .001), and this association remained significant in adjusted models for sociodemographic and vascular factors and *APOEε4* status (adjusted OR, 1.65; 95% CI, 1.33-2.20; *P* < .001). *APOEε4* carriers of both sexes had an approximately 4-fold greater odds of plaques. Significant 2-way interactions were found between sex, *APOEε4* status, race, and ancestry on CERAD scores. Black noncarriers (OR, 0.47; 95% CI, 0.34-0.67) and noncarriers of African ancestry (OR, 0.57; 95% CI, 0.43-0.76) were least likely to have high plaque burden, whereas this protection was weakened in ε4 carriers. No significant 3-way interaction was detected. Among individuals with a CERAD score of 2 or higher, female individuals were more likely than male individuals to reach Braak stage V-VI than male individuals (probability ratio, 1.25; 95% CI, 1.13-1.38; *P* = .002). Adding Braak stage to multivariable models attenuated the female-male difference in plaques and interaction of sex and plaque on CDR-SB was no longer significant.

**Conclusions and Relevance:**

The findings indicate that female sex, *APOEε4*, and both race and African ancestry were jointly associated with amyloid in this study population. Excess amyloid among women may partly explain their greater tau burden and steeper cognitive decline. These findings highlight the importance of incorporating sex, race, and ancestry into biomarker thresholds, risk stratification, and the design of preventive or disease-modifying trials for Alzheimer disease.

## Key Points

**Question:**

Do male and female individuals differ in neuritic plaque deposition, and does sex modify the influence of apolipoprotein E ε4, informant-reported race, or African genetic ancestry on plaque burden?

**Findings:**

In this cross-sectional study of 2268 autopsied adults, female sex was independently associated with greater neuritic plaque burden, especially White female individuals; among participants with moderate/frequent plaques, female individuals were more likely to reach high tau stages than male individuals, but sex did not modify the *APOEε4* effect. The association between *APOEε4* and neuritic plaque burden varied by race and African ancestry, with lower plaque burden observed in Black individuals and those with African ancestry only among ε4 noncarriers; among ε4 carriers, plaque burden did not differ by race or ancestry.

**Meaning:**

In this study, amyloid and tau burdens were higher in women, and ancestry modulated *APOEε4*-related risk, underscoring the need for sex- and ancestry-specific thresholds in Alzheimer disease risk assessment and targeted interventions.

## Teaser

This cross-sectional study evaluates associations of neuritic plaque burden and cognitive outcomes with sex, apolipoprotein E ε4 status, race, and ancestry.

## Introduction

Female individuals, particularly those from racially and ethnically minoritized groups, are disproportionately affected by dementia,^1,2,3^ yet the sociocultural factors underlying these disparities are poorly understood. Alzheimer disease (AD) is the most common cause of dementia worldwide. While numerous neuroimaging and several postmortem studies have investigated sex differences in AD hallmarks, findings on amyloid pathology are inconsistent compared to the more consistent evidence for sex differences in tau burden.^4,5,6,7^ Some studies report higher amyloid accumulation in female individuals,^6,8,9^ while others found no significant differences^1,10,11,12^ or, less commonly, greater amyloid burden in male individuals.^13,14^

Similarly, despite increasing recognition of racial disparities in AD prevalence, few studies have explored the intersection of race and sex with respect to amyloid pathology. Existing research has yielded conflicting results on amyloid burden and genetic risk factors, such as apolipoprotein E (*APOE*) genotype across different ethnic and racial groups.^15,16,17,18,19,20,21^

These inconsistencies likely reflect differences in study design and analytic approaches, as well as reliance on predominantly White cohorts with limited stratification by sex, race, or ancestry. Additionally, neuroimaging studies, primarily using amyloid positron emission tomography (PET), constitute the majority of investigations into sex differences but have limited sensitivity for detecting low amyloid levels during early or moderate disease stages, potentially overlooking subtle pathological changes.^22,23^ In contrast, neuropathological analyses offer greater sensitivity to low amyloid burdens but have been largely confined to convenience samples composed mainly of older White individuals or those with advanced dementia, restricting generalizability to diverse populations.^24,25^ To address this gap, we examined sex differences in amyloid pathology, their interactions with apolipoprotein E ε4 (*APOEε4*), race, and African ancestry and effects on cognition in a well-characterized, ethnically diverse, and population-based postmortem cohort, composed of Black and White participants. We also assessed sex differences in tau pathology across amyloid levels. Drawing on existing literature and prior findings from our group showing lower neuritic plaque burden in individuals with African ancestry,^15,19,26,27^ we hypothesized greater amyloid burden in female individuals, particularly Black female individuals, and relative protection from African ancestry, especially among male individuals.

## Methods

### Participants

The Biobank for Aging Studies at the University of São Paulo Medical School, São Paulo, Brazil, included 2418 postmortem brain donors, collected between April 2004 and March 2025. Ethical approval was obtained from the ethical committee of the University of Sao Paulo along with next-of-kin informed signed consent for brain donation. Detailed protocols, selection criteria, and variable definitions are published elsewhere.^26,28,29,30^ The study adhered to the Strengthening the Reporting of Observational Studies in Epidemiology (STROBE) reporting guideline.

### Clinical Assessment

Sociodemographic data (age, sex, education, race) were collected from the next of kin. The interviewer asked the next of kin to report the race of the deceased using the 5 standard Brazilian Institute of Geography and Statistics race categories^31^ (see the eMethods and eFigure 1 in Supplement 1 for full definitions and exclusions). For the analysis, race was categorized as Black or White. Black and Pardo groups were combined because of shared experiences of racism and health disparities, consistent with prior Brazilian research,^32^ and because DNA-based ancestry analyses in a subset of this population showed similar percentages of European ancestry in both groups.^19^ Asian individuals were excluded due to small sample size (n = 49), and no individuals were classified as Indigenous.

Vascular risk factors and lifestyle variables were assessed with a semistructured questionnaire and coded as binary indicators, and body mass index (BMI) measured before autopsy was analyzed as a continuous variable. Detailed definitions and measurement procedures are provided in the eMethods in Supplement 1.

Cognitive functional status was assessed by informant-rated Clinical Dementia Rating (CDR) score within 3 months of death, classified as cognitively normal (CDR-Global = 0) or impaired (CDR-Global ≥0.5). The CDR Sum of Boxes (CDR-SB) was used as a continuous measure of cognitive severity.

### Genetic Analysis

*APOE *genotyping was performed using real-time polymerase chain reaction^33^; participants were classified as *APOEε4* carriers (≥1 ε4 allele) or noncarriers (ε2/ε2, ε2/ε3, ε3/ε3), and those with ε2/ε4 were excluded (n = 30). Global trihybrid continental ancestry was estimated from 47 ancestry-informative markers^34^ and analyzed both dichotomously (African ancestry ≥2% vs <2%) and continuously (0–1); full genotyping and ancestry procedures are detailed in the eMethods in Supplement 1.

### Neuropathology

Brain tissue processing followed standardized Biobank for Aging Studies protocols and procedures.^15,30^ AD pathological changes were evaluated using Braak and Braak staging for neurofibrillary tangles,^35^ the Consortium to Establish a Registry for Alzheimer’s Disease (CERAD) neuropathological score for neuritic plaque,^36^ and Thal amyloid phases,^37^ which were also integrated into the ABC score (A, amyloid β deposits [Thal phase]; B, neurofibrillary tangle stage [Braak stage]; C, neuritic plaque score [CERAD]), following the National Institute on Aging and Alzheimer’s Association guidelines, to provide an AD neuropathologic change level.^38^ Lewy body disease was classified using Braak staging for Parkinson disease.^39^ Lacunar infarcts, small vessel disease (including arteriosclerosis and lipohyalinosis), and cerebral amyloid angiopathy were classified based on location, extent, and severity. Additional age-related neuropathologies included limbic-predominant age-related TDP-43 encephalopathy and hippocampal sclerosis. CERAD score (0-3; none, sparse, moderate, frequent), Thal amyloid phase (0-5), ABC score (0-3), and AD Braak stage (0-II, III-IV, V-VI) were treated as categorical ordinal variables. Other neuropathologies were coded as absent or present. For more details, see the eMethods in Supplement 1 and previous publications.^15,29,30^

### Statistical Analyses

Demographic, clinical, and pathological characteristics were summarized using descriptive statistics and compared by sex. Continuous variables were reported as medians with IQRs and compared using Mann-Whitney *U*/Wilcoxon tests. Categorical variables were summarized as counts and percentages and compared using Pearson χ^2^. Associations between sex and amyloid pathology were evaluated using ordinal (and, in sensitivity analyses, ordinal and multinomial) logistic regression with CERAD neuritic plaque burden and Thal amyloid phase as outcomes (see eTables 1-4 in Supplement 1 for full model results). Models were progressively adjusted for demographic characteristics (age, race, education), *APOEε4* status, vascular risk factors, and Braak stage to account for tau burden and disease severity; in a subset with genetic data, African ancestry, analyzed dichotomously (≥2% vs <2%), was used instead of informant-reported race and was also modeled as a continuous variable in a sensitivity analysis.

We further examined whether sex-amyloid associations were modified by race, African ancestry, and *APOEε4 *using interaction terms and categorical combinations of sex with race, African ancestry, and *APOEε4*, with omnibus and post hoc tests reported in eTables 5-6 in Supplement 1. Linear regression models related sex and amyloid pathology to cognition (CDR-SB score), with sequential adjustment for demographic characteristics, *APOEε4*, vascular risk factors, Braak stage, and copathologies, and sensitivity analyses used dichotomized CERAD neuropathological scores and ordinal CDR-Global score. All analyses used a 2-sided α = .05, with Bonferroni correction for post hoc pairwise comparisons. Full model specifications, interaction structures, sensitivity analyses, and diagnostics are provided in the eMethods and eResults in Supplement 1.

## Results

### Sex Differences in Demographic, Clinical, and Risk Factor Profiles

Among 2268 participants (median [IQR] age, 74.8 [63.8-83.3] years; 1152 [51% male] and 1116 [49%] female; 802 [35%] Black and 1466 [65%] White), female individuals were older, had higher prevalence of cognitive impairment and hypertension, and had less school attainment and rates of smoking and alcohol use than male individuals (Table 1).

**Table 1.** Demographic and Clinical Characteristics, Stratified by Sex (N = 2268)^a^

Characteristic	No.^b^	No. (%)			*P* value
		Total (N = 2268)	Male (n = 1152 [51%])	Female (n = 1116 [49%])	
Age, median (IQR), y	2268	74.8 (63.8-83.3)	71.4 (61.3-80.1)	78.5 (68.1-85.4)	<.001
Race^c^					
Black	2268	802 (35)	413 (36)	389 (35)	.63
White		1466 (65)	739 (64)	727 (65)	
Education, median (IQR), y	2268	4.0 (2.0-8.0)	4.0 (4.0-8.0)	4.0 (1.0-5.0)	<.001
CDR sum of boxes, median (IQR)	2268	0.0 (0.0-4.0)	0.0 (0.0-1.0)	0.0 (0.0-10.0)	<.001
CDR global					
0	2268	1503 (66)	842 (73)	661 (59)	<.001
0.5		208 (9)	105 (9)	103 (9)	
≥1		557 (25)	205 (18)	352 (32)	
Dichotomized African ancestry					
No significant African ancestry (<2%)	578	137 (23)	66 (24)	71 (24)	>.99
African ancestry (≥2%)		441 (76)	213 (76)	228 (76)	
*APOEε4* carrier	1460	439 (30)	223 (30)	216 (30)	>.99
*APOE* allele^d^					
ε2/ε2	1460	8 (1)	4 (1)	4 (1)	.74
ε2/ε3		135 (9)	75 (10)	60 (8)	
ε3/ε3		878 (60)	439 (59)	439 (61)	
ε3/ε4		391 (27)	201 (27)	190 (26)	
ε4/ε4		48 (3)	22 (3)	26 (4)	
Vascular risk factors					
Hypertension, yes	2263	1456 (64)	684 (60)	772 (69)	<.001
Diabetes, yes	2264	666 (29)	326 (28)	340 (31)	.27
Coronary artery disease, yes	2264	440 (19)	232 (20)	208 (19)	.37
Cardiac failure, yes	2264	376 (17)	189 (16)	187 (17)	.87
Dyslipidemia, yes	2265	317 (14)	157 (14)	160 (14)	.63
Stroke, yes	2265	303 (13)	152 (13)	151 (14)	.85
BMI, median (IQR)	2265	22.8 (19.5-25.9)	23.1 (20.0-25.6)	22.7 (19.3-26.1)	.51
Smoking, yes^e^	2218	1115 (50)	710 (63)	405 (37)	<.001
Alcohol					
Never/sometimes	2197	1568 (71)	637 (57)	931 (87)	<.001
Current heavy use		190 (9)	158 (14)	32 (3)	
Former heavy use		439 (20)	332 (29)	107 (10)	

Abbreviations: *APOE*, apolipoprotein E; BMI, body mass index (calculated as weight in kilograms divided by height in meters squared); CDR, Clinical Dementia Rating scale.

^a^ Continuous variables were compared using Mann-Whitney U/Wilcoxon tests. Categorical variables were compared using the Pearson χ^2^ test.

^b^ Number of nonmissing values.

^c^ Race was reported by next of kin using standard Brazilian Institute of Geography and Statistic categories and analyzed as Black (Black plus Pardo) vs White; Asian individuals were excluded due to small numbers, and no individuals were classified as Indigenous.

^d^ Individuals with ε2/ε4 (n = 30) were excluded from the analysis.

^e^ Smoking (yes) current or former use combined groups.

### Sex Differences in AD Pathology

**Figure 1.**
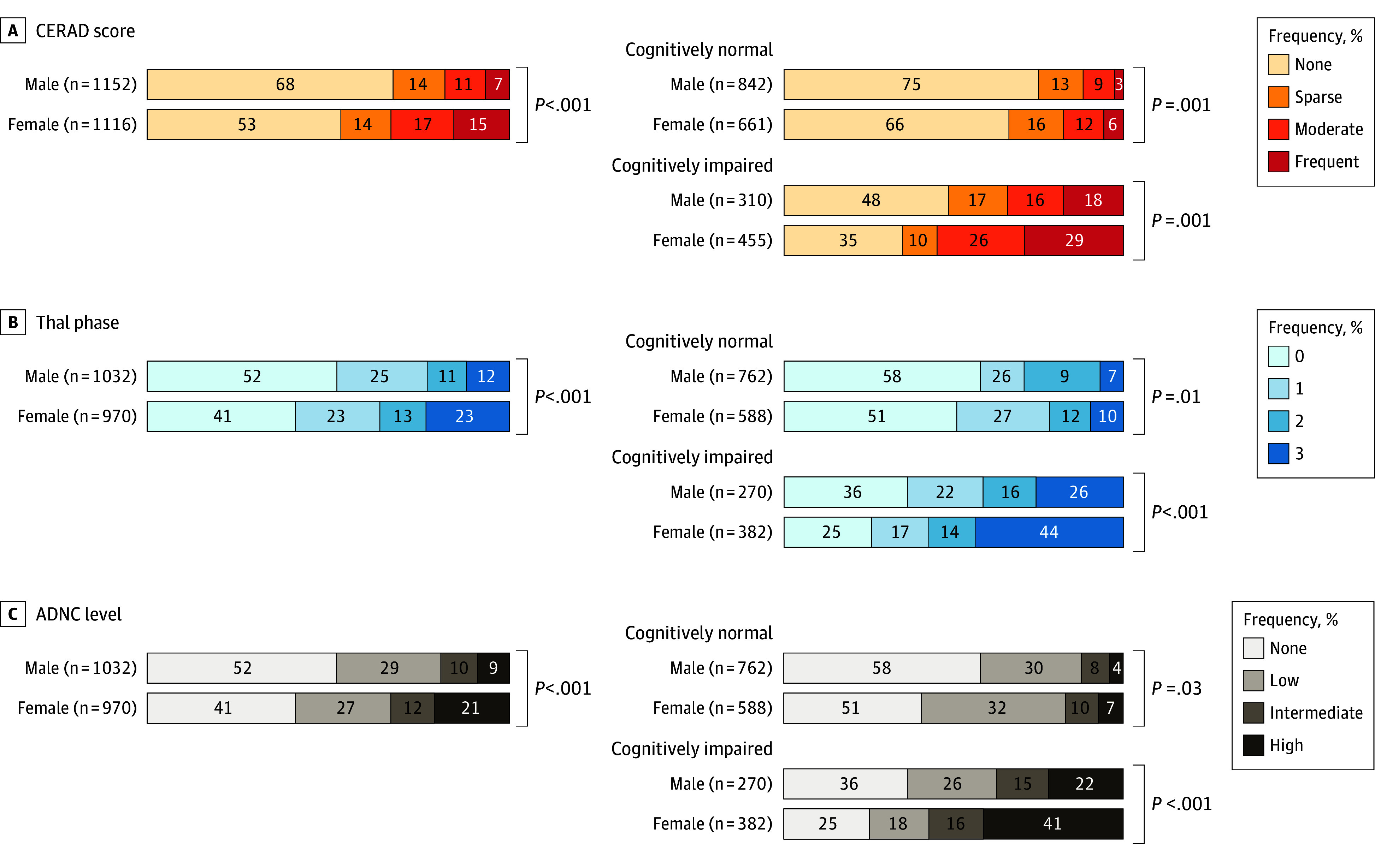
Bar Graphs of the Distribution of Alzheimer Disease (AD) Consortium to Establish a Registry for Alzheimer’s Disease (CERAD) Scores, Thal Phases, and AD Neuropathologic Change (ADNC) Levels Across Sex Groups The figure shows sex differences in Alzheimer disease neuropathology across the full cohort and stratified by cognitive status (cognitively normal: Clinical Dementia Rating-Global score of 0; cognitively impaired: Clinical Dementia Rating-Global score >0). Comparisons were conducted using χ^2^ tests to evaluate differences between male and female individuals in the overall sample as well as within the cognitively normal and cognitively impaired subgroups. Separate distributions are shown for cognitively normal male individuals, cognitively normal female individuals, cognitively impaired male individuals, and cognitively impaired female individuals. Female individuals showed significantly higher Thal phases, CERAD scores, and ADNC scores compared to male individuals in all analyses (eTable 7 in Supplement 1). These sex differences remained significant when stratified by clinical status (cognitively normal and cognitively impaired), suggesting consistent patterns of greater amyloid pathology burden in female individuals across disease stages.

**Figure 2.**
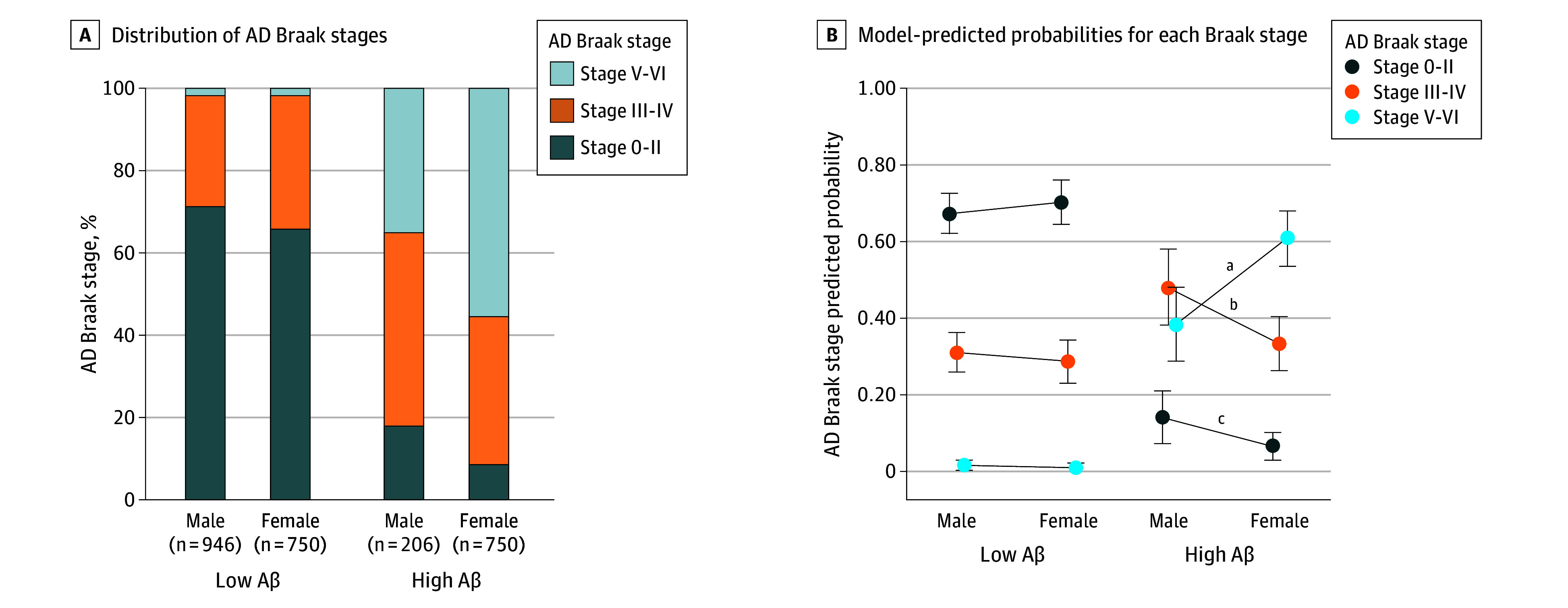
Bar Graph and Dot Plot of Sex and Amyloid Group Differences According to the Braak Stages of Neurofibrillary Tangles A, The stacked bar plot shows the distribution of AD Braak stages (0-II, III-IV, V-VI) stratified by combined sex-amyloid groups. Low amyloid was defined as none or sparse Consortium to Establish a Registry for Alzheimer’s Disease (CERAD) scores, and high amyloid as moderate or frequent CERAD scores. B, The dot plot shows model-predicted probabilities (with 95% CIs) for each Braak stage, derived from multinomial logistic regression adjusted for age, race, apolipoprotein E ε4 (*APOEε4*), and sex-amyloid status. Race was reported by next of kin using standard Brazilian Institute of Geography and Statistic categories and analyzed as Black (Black plus Pardo) vs White; Asian individuals were excluded due to small numbers, and no individuals were classified as Indigenous. ^a^Female individuals had a 25% higher probability of Braak stage V-VI compared with male individuals (95% CI, 13-38, Bonferroni-adjusted *P* = .002). ^b^Female individuals had a 14% lower probability of Braak stage III-IV compared to male individuals (95% CI, 4-22, *P* = .02). ^c^Female individuals had a 7% lower probability of Braak stage 0-II compared to male individuals (95% CI, 1-13, *P* = .04).

Group comparisons showed female individuals had higher proportions of high CERAD neuropathological scores, advanced Thal phases, and high AD neuropathologic change ratings compared to male individuals, regardless of cognitive status (Figure 1; eTable 7 in Supplement 1). In ordinal regression, female sex was associated with higher CERAD neuropathological scores and Thal phases compared with male sex, including in models adjusted for age, education, race or African ancestry, *APOEε4*, and cardiovascular risk factors. Female individuals had higher CERAD scores (unadjusted odds ratio [OR], 1.97; 95% CI, 1.67-2.29; *P* < .001), and this association remained significant in fully adjusted models (OR, 1.65; 95% CI, 1.33-2.20; *P* < .001). Similarly, female individuals had higher Thal phases, with associations that remained significant in fully adjusted models (OR, 1.61; 95% CI, 1.27-1.98; *P* < .001) (Table 2). These associations were attenuated and nonsignificant after adjusting for Braak stage. Similar patterns were observed in analyses restricted to participants with cognitive impairment and in models treating CERAD as categorical or African ancestry as continuous (eTables 2-4 in Supplement 1). Given the attenuation of sex differences in amyloid burden after adjusting for Braak stage, we conducted complementary analyses to examine whether these differences persist within Braak stage strata and whether tau pathology differs by sex at comparable amyloid levels (eMethods an eTable 1 in Supplement 1; Figure 2). In models with CERAD score as the outcome, stratified by Braak stage, sex differences in amyloid burden were most pronounced at advanced Braak stages (V-VI). With progressive adjustment, the odds ratios increased from 1.85 (95% CI, 1.18-3.32; *P* = .02) to 2.53 (95% CI, 1.35-9.11; *P* = .009) in the fully adjusted model, whereas differences at earlier stages (0-II, III-IV) were small and often nonsignificant (eTable 1 in Supplement 1). In sex-amyloid–stratified models predicting Braak stage, among individuals with high amyloid burden (moderate or frequent CERAD neuritic plaque scores), female individuals were more likely than male individuals to reach advanced Braak stages V-VI, both in observed proportions (55% vs 35%) and predicted probabilities (probability ratio 1.25; 95% CI, 1.13-1.38; *P* = .002), with no sex differences observed at lower amyloid levels (Figure 2).

**Table 2.** Association Between Sex and Neuropathologic Outcomes: Consortium tof Establish a Registry for Alzheimer’s Disease (CERAD) Neuropathological Score and Thal Amyloid-β Phase (N = 2268)

Metric	Female sex^a^									
	Model 1^b^		Model 2^c^		Model 3^d^		Model 4^e^		Model 5^f^	
	OR (95% CI)^g^	*P* value	OR (95% CI)^g^	*P* value	OR (95% CI)^g^	*P* value	OR (95% CI)^g^	*P* value	OR (95% CI)^g^	*P* value
**AD CERAD score (0-3)**										
Models adjusted for race	1.97 (1.67-2.29)	<.001	1.44 (1.23-1.71)	<.001	1.61 (1.31-2.05)	<.001	1.65 (1.33-2.20)	<.001	1.2 (0.94-1.55)	.16
No.	2268	NA	2268	NA	1460	NA	1443	NA	1443	NA
Models adjusted for African ancestry	2.00 (1.52-2.81)	<.001	1.44 (1.05-2.12)	.04	1.63 (1.17-2.44)	.008	1.71 (1.26-2.72)	.006	1.39 (0.97-2.34)	.12
No.	578	NA	578	NA	578	NA	568	NA	568	NA
**Thal phase (0-5)**										
Models adjusted for race	1.76 (1.51-2.08)	<.001	1.33 (1.12-1.57)	.001	1.56 (1.34-2.12)	<.001	1.61 (1.27-1.98)	<.001	1.21 (0.97-1.56)	.10
No.	2002	NA	2002	NA	1311	NA	1296	NA	1296	NA
Models adjusted for African ancestry	2.00 (1.46-2.98)	<.001	1.54 (1.09-2.23)	.02	1.84 (1.34-2.85)	.002	1.95 (1.29-3.24)	.001	1.61 (1.03-2.80)	.03
No.	447	NA	447	NA	447	NA	439	NA	439	NA

Abbreviations: NA, not applicable; OR, odds ratio.

^a^ Reference: male sex. Results from ordinal logistic regression analyses assessing the association between sex and Alzheimer disease CERAD scores (none, sparse, moderate, frequent) and Thal phase (0-5) as an outcome.

^b^ Model 1: unadjusted ordinal logistic regression model.

^c^ Model 2: ordinal logistic regression model adjusted for age, education, and reported race or African ancestry.

^d^ Model 3: ordinal logistic regression model adjusted for age, education, race or African ancestry, and apolipoprotein E ε4 (*APOEε4*).

^e^ Model 4: ordinal logistic regression model adjusted for age, education, race or African ancestry, *APOEε4*, and cardiovascular risk factors (defined as the presence [yes/no] of hypertension, diabetes, dyslipidemia, coronary artery disease, heart failure, stroke, or smoking; body mass index was included as a continuous variable).

^f^ Model 5: ordinal logistic regression model adjusted for age, education, race or African ancestry, *APOEε4*, cardiovascular risk factors (defined as the presence [yes/no] of hypertension, diabetes, dyslipidemia, coronary artery disease, heart failure, stroke, or smoking; body mass index was included as a continuous variable), and Alzheimer disease Braak stages.

^g^ ORs for sex from the ordinal regression analysis are presented, with 95% CIs estimated from 1000 bootstrap samples and corresponding *P* values.

### Interactions Between Sex, *APOEε4*, and Race/Ancestry on Neuritic Plaque Burden

**Figure 3.**
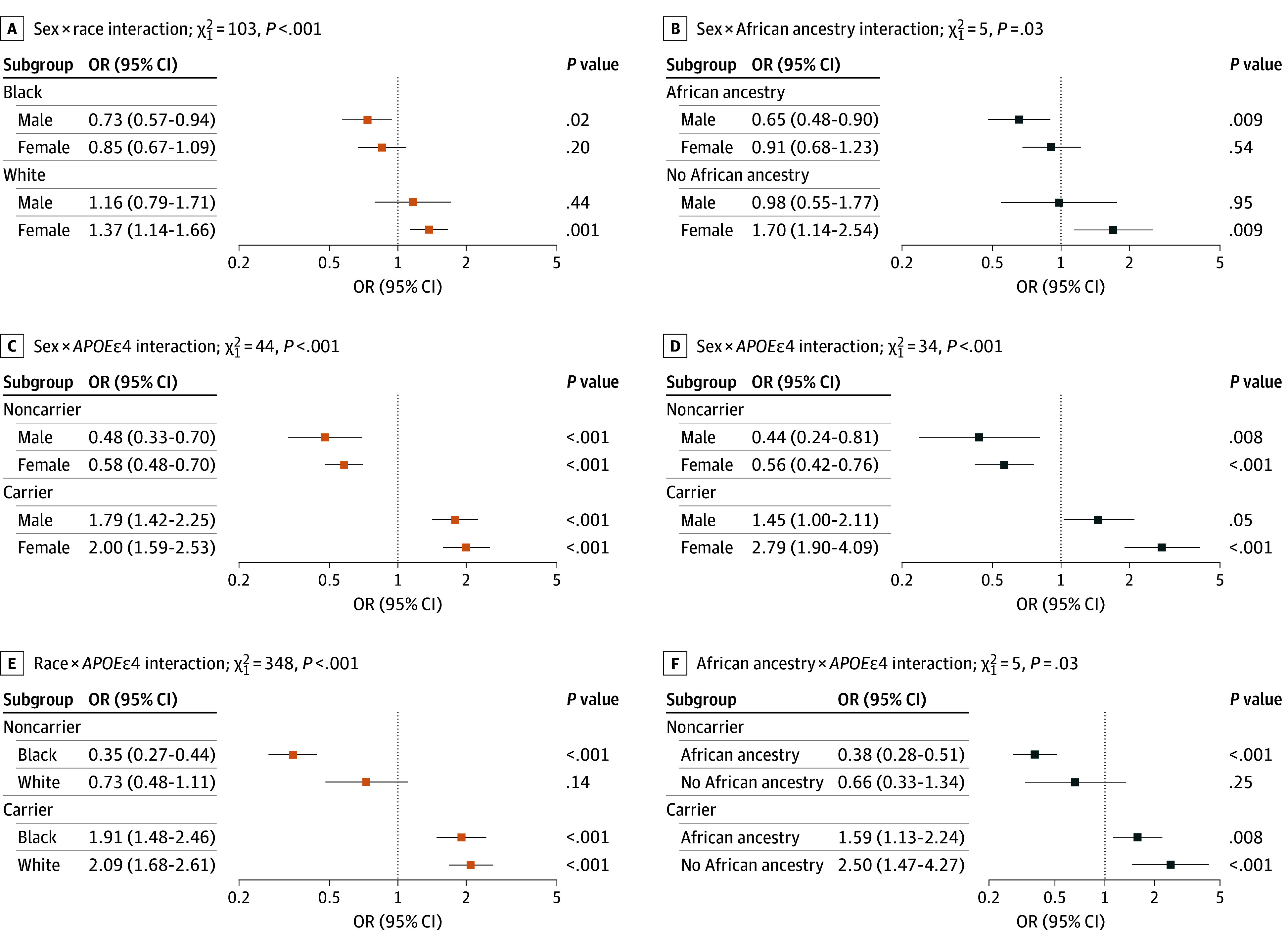
Forest Plots Showing 2-Way Interactions of Sex, Race or African Genetic Ancestry, and Apolipoprotein E ε4 (*APOEε4*) in Relation to Consortium to Establish a Registry for Alzheimer’s Disease (CERAD) Neuritic Plaque Burden The figure displays odds ratios (ORs) and 95% CIs from ordinal logistic regression models predicting CERAD neuropathological scores. Omnibus χ^2^ statistics and associated *P* values above each panel indicate the significance of the 2-way interaction terms. Panels in the left column correspond to model A using reported race (n = 1460); panels in the right column correspond to model B using genetically inferred African ancestry (n = 578). Model A includes sex, age, race (Black/White), *APOEε4* status (carrier vs noncarrier), and Braak stage. Model B includes sex, age, genetically inferred African ancestry, *APOEε4* status (carrier vs noncarrier), and Braak stage. ORs were derived using deviation (sum-to-zero) coding, where each value reflects the stratified subgroup’s deviation from the grand mean rather than from a reference group. Post hoc comparisons from these interaction models and stratified subgroup analyses are presented in eTable 5 in Supplement 1. Results from corresponding 3-way interaction models (sex × *APOEε4* × race/African ancestry) are provided in eFigure 2 and eTable 5 in Supplement 1. Race was reported by next of kin using standard Brazilian Institute of Geography and Statistic categories and analyzed as Black (Black plus Pardo) vs White; Asian individuals were excluded due to small numbers, and no individuals were classified as Indigenous.

Details are shown in Figure 3 and eTable 5 in Supplement 1. The sex × race interaction was driven by the highest plaque burden in White female individuals and the lowest in Black male individuals, with Black participants showing lower odds of greater CERAD neuropathological scores than Whites across sexes. In the sex × African ancestry interaction, female individuals without African ancestry had the highest burden and male individuals with African ancestry the lowest; sex differences within race or ancestry strata were modest and generally nonsignificant after adjustment. Overall, Black race and African ancestry were associated with lower neuritic plaque burden, independent of sex.

The sex and *APOEε4* interaction was statistically significant and indicated a roughly 4-fold higher odds of greater CERAD neuropathological scores in carriers vs noncarriers for both sexes but with only modest differences in effect size between female and male individuals and overlapping confidence intervals. The race and *APOEε4* interaction was significant (χ^2^_1_ = 349; *P* < .001), indicating differential effects of *APOEε4* by race. White *APOEε4* carriers had 2.9-fold higher odds of greater CERAD neuropathologic scores than White noncarriers, whereas Black carriers had 5.5-fold higher odds than Black noncarriers. Using White noncarriers as the reference, Black noncarriers had substantially lower odds of high plaque burden (OR, 0.47; 95% CI, 0.34-0.67), with no significant difference between Black and White carriers.

In African ancestry–adjusted models (χ^2^_1_ = 5; *P* = .03), noncarriers with African ancestry had lower odds than those without (OR, 0.57; 95% CI, 0.43-0.76; *P* < .001), while among *APOEε4* carriers, odds did not differ by ancestry. Three-way interactions of sex, race, African ancestry, and *APOEε4* were not significant. *APOEε4* consistently predicted higher neuritic plaque burden across subgroups, and some subgroup estimates had wide confidence intervals, reflecting limited power in smaller strata (Figure 3; eFigure 2 and eTable 5 in Supplement 1).

### Intersectional Effects of Race, African Ancestry, Sex, and *APOEε4* on Amyloid Pathology

Exploratory models combining sex, race, African ancestry, and *APOEε4* suggested the lowest plaques in Black men with African ancestry and the highest in White women without African ancestry, especially among *APOEε4* noncarriers (eFigure 3 and eTable 6 in Supplement 1). However, some subgroups had small sample sizes, resulting in imprecise estimates with wide confidence intervals, which should be interpreted with caution.

### Sex-Specific Associations Between Neuritic Plaques and Cognitive Function

We examined whether sex modified the association between CERAD neuropathological score and cognition (CDR-SB). Sex alone was not related to cognition, whereas higher CERAD neuropathological scores predicted worse function (eTable 8 in Supplement 1). A sex × CERAD score interaction remained significant after adjustment for demographic characteristics, *APOEε4*, and cardiovascular risk factors, but became nonsignificant after controlling for Braak stage and copathologies; CERAD neuropathological scores remained a strong predictor in all models. Three-way interactions of sex, race, African ancestry, and *APOE* were nonsignificant, and sensitivity analyses using dichotomized CERAD or CDR-Global yielded similar results (eTables 9-10 in Supplement 1). In CDR-Global–stratified analyses, female individuals had higher odds of greater neuritic plaque burden at CDR 3 and CDR 0, although the latter association was no longer significant after full adjustment (eTable 11 in Supplement 1).

## Discussion

In this cross-sectional study, we demonstrated that sex, informant-reported race, genetically defined African ancestry, and *APOEε4* genotype were associated with amyloid pathology burden in brains, providing new insights into biologically meaningful heterogeneity in AD neuropathology.

### Sex Differences and Neuropathological Sensitivity

Previous studies on sex differences in amyloid pathology have yielded mixed results. Several studies reported higher plaque burden in female individuals.^6,8,9^ However, primarily imaging studies using amyloid PET have found inconsistent or no sex differences, likely due to limited amyloid PET sensitivity for detecting low to moderate amyloid levels.^1,10,11,12,13,14^ Our neuropathological analysis, with its superior sensitivity, confirms that female individuals have greater odds of higher burden of amyloid deposition than male individuals, helping to reconcile discrepancies across earlier reports. Sex differences in neuritic plaque burden persisted after adjusting for demographic, clinical, and vascular covariates but were attenuated when tau pathology was added, suggesting that part of the sex differences in amyloid plaques burden may reflect the well-documented differences in tau burden.^4,5,6,7^ In Braak stage–stratified sensitivity analyses, women exhibited greater amyloid burden than men only at advanced tau stages (Braak V-VI), while no significant sex differences were observed in earlier Braak stages, suggesting that sex-related differences in amyloid pathology emerge predominantly in the context of late-stage tau pathology and are not fully explained by demographic characteristics, *APOEε4*, or cardiovascular risk factors. Moreover, the observed sex-specific pattern in tau pathology that emerges alongside elevated amyloid burden, which may contribute to the more severe AD pathology, helps explain previously observed differences in disease severity and progression in female individuals.^5,40,41,42^ In sensitivity analyses stratified by global cognitive status, female individuals showed higher neuritic plaque burden at the severe dementia level (CDR 3) and a nonsignificant trend toward higher CERAD neuropathological scores in the cognitively unimpaired group (CDR 0) after adjustment. However, given the ceiling effects inherent to CDR 3, the loss of significance at CDR 0 in fully adjusted models, and limited power in intermediate CDR strata, these findings do not permit firm conclusions regarding sex differences in resilience to amyloid^43,44^ and should instead be viewed as preliminary patterns that require confirmation in larger, better-powered samples across the full range of cognitive impairment.

In our cohort, women were on average older than men, in line with well-established sex differences in longevity.^45,46,47^ Greater age may partially underlie the higher amyloid burden and more severe cognitive impairment observed in female individuals. Conversely, men who reach very advanced ages likely represent a selectively surviving group, which could shape the neuropathologic profile we observe. In addition, large-scale studies indicate that men with dementia experience higher mortality than women^48^ further raising the possibility of survivorship bias. Although we adjusted for age in all models, we cannot entirely rule out residual bias related to differential survival.

### Race, African Ancestry, and the Biological Dimension of Disparities

While racial categories predominantly reflect social constructs encompassing environmental and cultural factors, genetic ancestry also captures inherited biological variation influencing disease susceptibility. Our data indicated that genetically determined African ancestry was associated with significantly lower odds of neuritic plaque deposition, particularly in male individuals. Notably, these associations were slightly stronger for African ancestry than for race and remained significant after adjusting for Braak stage and overall disease severity. This persistence signals that African ancestry may confer biological variation in amyloid burden beyond social determinants commonly indexed by race. This affirms our previous neuropathological-genetic analysis^19,26^ and consistent with prior genetic epidemiology studies that reported protective effects of African ancestry on amyloid accumulation and AD risk.^49^ For example, Rajabli et al^50^ found that African-derived genetic variants influenced AD risk and neuropathological phenotypes, underscoring the importance of incorporating genetic ancestry into AD research to disentangle biological heterogeneity from environmental or social influences.

### *APOEε4* and Its Ancestry-Modulated Effects

The *APOEε4* allele remains the strongest and most replicated genetic risk factor for late-onset AD. As expected, *APOEε4* carriers in our cohort had markedly elevated neuritic plaque burden regardless of sex, race, or ancestry group. Although the sex × *APOEε4* interaction reached statistical significance, the effect size was modest and confidence intervals overlapped between sexes, indicating broadly similar *APOEε4*-related increases in neuritic plaque burden in women and men. Prior autopsy and biomarker studies have reported inconsistent evidence for sex-specific *APOEε4* effects on amyloid deposition, suggesting that differences in cohort age, sample composition, and sensitivity to early pathology may contribute to these discrepancies.^13,18,51,52^ In our relatively younger, population-based autopsy cohort, observed sex differences were likewise modest, consistent with this heterogeneous prior literature. A key observation is the attenuation of *APOEε4*’s effect magnitude among individuals with greater African ancestry in concordance with our earlier studies with a smaller sample size. This evidence of a gene–ancestry interaction aligns with epidemiological data suggesting *APOEε4* confers lower AD risk in African descent populations, potentially due to genetic modifiers or gene–environment interactions that remain to be fully elucidated.^49^

Our findings highlight the limitations of applying risk estimates derived predominantly from European ancestry populations to admixed or underrepresented groups. They also signal the need for precision medicine approaches integrating race and genetic ancestry to improve risk prediction and therapeutic targeting.

### Intersectionality and Clinical Relevance

By examining sex, race, ancestry, and *APOEε4* together, we found complex patterns: White female individuals consistently had the highest amyloid burden, whereas Black male individuals with high African ancestry had the lowest. These differences argue against simplified sex- or race-based assumptions and highlight the value of intersectional approaches. Clinically, our findings align with trials reporting sex differences in response to amyloid-lowering therapies, with female individuals often showing reduced benefit, possibly owing to greater tau pathology or more advanced disease.^53,54,55^ In our cohort, female individuals with high amyloid also had greater tau burden, which may further blunt treatment response and underscores the need to consider sex and ancestry in trial design and stratification to better match therapeutics to AD’s heterogeneous biology.

### Strengths and Limitations

Strengths of this study include a large, population-based sample representative of the admixed Brazilian population, detailed neuropathological assessments enabling sensitive detection of early pathology, and integration of risk factors, vascular pathology, social race, and genetic ancestry. This design allows comprehensive evaluation of contributors to AD pathology in a diverse setting, reducing biases of clinic-based or homogeneous cohorts and improving generalizability. The study was adequately powered to detect subtle sex differences in amyloid pathology in both the overall cohort and participants with AD-related cognitive impairment. Inclusion of both informant-identified race and quantified African ancestry provided a nuanced view of social and biological influences on AD pathology. Of note, our genetic analyses captured only continental-level ancestry and cannot resolve subcontinental components, such as Iberian vs other European contributions. Limited sample sizes for some interactions restricted the power to detect subtle effects. Also, in contemporary Brazilian demographic usage, most admixture classified as Pardo reflects combinations of European and African (Black and White) ancestries; however, we acknowledge that historically the term has also encompassed mixed European, African, and Indigenous ancestries. Analyses of *APOEε2* were not undertaken because of limited sample size and sparse representation across sex, race, ancestry, and cognitive strata; this question will require larger, multicohort samples to be adequately addressed. The cross-sectional postmortem design precludes longitudinal inferences regarding progression and causality. Furthermore, while we address biological and social constructs, additional factors, such as environment, socioeconomic status, and epigenetics, likely interact to influence AD disparities and merit future study. Although prior work in this cohort suggests that African ancestry, including local African ancestry at *APOE*, is associated with lower odds of severe neuritic plaque burden and that major copathologies are unlikely to fully explain worse cognitive outcomes at comparable AD burden, the broader interplay among sex, *APOEε4*, ancestry, and copathologies remains unclear.^15,19^ Potential interactions of sex and *APOEε4* with vascular and other non-AD copathologies across ancestry groups were not assessed here and remain an important target for future, well-powered multicohort studies.

## Conclusions

Results from this study demonstrated that sex, race, genetically defined African ancestry, and *APOEε4* genotype were jointly associated with amyloid pathology severity. Our findings underscore the importance of neuropathology for refining biomarker interpretation. Incorporating ancestry and sex into biomarker development, clinical trial design, and therapeutic strategies is critical to reducing disparities and optimizing outcomes. Future longitudinal studies integrating neuropathology, multiomics, neuroimaging, and sociocultural data across diverse populations will be key to advancing equitable precision medicine.

## Notes

Supplement 1. eMethods eFigure 1. Flowchart of the Study Participants eResults eTable 1. Sensitivity analyses examined the association between sex and amyloid deposition, using CERAD Neuropathological scores and Thal phase measures, stratified by Braak stage subgroups eTable 2. Association between sex and amyloid deposition evaluated using the CERAD neuropathological scores and Thal Phase staging in individuals with ancestry data (n=578) eTable 3. Association between sex and CERAD Neuropathological score and Thal Phase in participants with Alzheimer’s disease (AD) and cognitive impairment (n=447) eTable 4. Association between sex and neuropathologic outcomes, CERAD neuropathological score as a categorical variable eTable 5. Summary of omnibus chi-square statistics (χ^2^) and post hoc comparisons from ordinal logistic regression models testing Sex–Race–APOEε4 interactions and stratified subgroup models predicting CERAD neuropathological score eFigure 2. Three-way interactions of sex, race/ancestry, and APOEε4 status on CERAD neuropathological scores. eTable 6. Adjusted pairwise comparisons from ordinal logistic regression models of stratified Sex–Race–AFR and APOEε4–Race–AFR subgroups predicting CERAD neuropathological score eFigure 3. Associations of Sex–Race–AFR and APOEε4–Race–AFR Subgroups with CERAD neuropathological scores. eTable 7. Neuropathological changes stratified by sex (n=2,268) eTable 8. Association between amyloid pathology and cognitive abilities evaluated using Clinica Dementia Rating Sum of Boxes (CDR-SB) considering Sex and Amyloid interactions (n=2,268) eTable 9. Association of sex and amyloid pathology with cognitive abilities (CDR-SB) considering interactions between sex and amyloid (n=2,268) eTable 10. Association of sex and amyloid pathology with cognitive abilities (CDR-Global) considering interactions between sex and amyloid eTable 11. Sensitivity analysis of the association between sex and amyloid deposition evaluated using the CERAD Neuropathological score across participants matched by functional cognitive level eReferences

Supplement 2. Data sharing statement
