# Supplementary material for: Sex Differences in Amyloid Pathology by Race, Ancestry, and Apolipoprotein E ε4 in an Admixed Autopsy Sample
Source: JAMA Neurol. 2026 Feb 23;83(4):392–401. doi: 10.1001/jamaneurol.2026.0054 (PMC12931469; doi:10.1001/jamaneurol.2026.0054)
Supplement: Supplement 1. — eMethods eFigure 1. Flowchart of the Study Participants eResults eTable 1. Sensitivity analyses examined the association between sex and amyloid deposition, using CERAD Neuropathological scores and Thal phase measures, stratified by Braak stage subgroups eTable 2. Association between sex and amyloid deposition evaluated using the CERAD neuropathological scores and Thal Phase staging in individuals with ancestry data (n=578) eTable 3. Association between sex and CERAD Neuropathological score and Thal Phase in participants with Alzheimer’s disease (AD) and cognitive impairment (n=447) eTable 4. Association between sex and neuropathologic outcomes, CERAD neuropathological score as a categorical variable eTable 5. Summary of omnibus chi-square statistics (χ2) and post hoc comparisons from ordinal logistic regression models testing Sex–Race–APOEε4 interactions and stratified subgroup models predicting CERAD neuropathological score eFigure 2. Three-way interactions of sex, race/ancestry, and APOEε4 status on CERAD neuropathological scores. eTable 6. Adjusted pairwise comparisons from ordinal logistic regression models of stratified Sex–Race–AFR and APOEε4–Race–AFR subgroups predicting CERAD neuropathological score eFigure 3. Associations of Sex–Race–AFR and APOEε4–Race–AFR Subgroups with CERAD neuropathological scores. eTable 7. Neuropathological changes stratified by sex (n=2,268) eTable 8. Association between amyloid pathology and cognitive abilities evaluated using Clinica Dementia Rating Sum of Boxes (CDR-SB) considering Sex and Amyloid interactions (n=2,268) eTable 9. Association of sex and amyloid pathology with cognitive abilities (CDR-SB) considering interactions between sex and amyloid (n=2,268) eTable 10. Association of sex and amyloid pathology with cognitive abilities (CDR-Global) considering interactions between sex and amyloid eTable 11. Sensitivity analysis of the association between sex and amyloid deposition evaluated using the CERAD Neuropathological sco [file jamaneurol-e260054-s001.pdf]

Supplemental Online Content

Abu Raya M, Suemoto CK, Paes VR, et al. Sex differences in amyloid pathology by race, ancestry, and apolipoprotein E ε4 in an admixed autopsy sample. *JAMA Neurol*. Published online February 23, 2026. doi:10.1001/jamaneurol.2026.0054

eMethods
eFigure 1. Flowchart of the Study Participants
eResults
eTable 1. Sensitivity analyses examined the association between sex and amyloid deposition, using CERAD Neuropathological scores and Thal phase measures, stratified by Braak stage subgroups
eTable 2. Association between sex and amyloid deposition evaluated using the CERAD neuropathological scores and Thal Phase staging in individuals with ancestry data (n=578)
eTable 3. Association between sex and CERAD Neuropathological score and Thal Phase in participants with Alzheimer's disease (AD) and cognitive impairment (n=447)
eTable 4. Association between sex and neuropathologic outcomes, CERAD neuropathological score as a categorical variable
eTable 5. Summary of omnibus chi-square statistics (χ^2) and post hoc comparisons from ordinal logistic regression models testing Sex–Race–APOEε4 interactions and stratified subgroup models predicting CERAD neuropathological score
eFigure 2. Three-way interactions of sex, race/ancestry, and APOEε4 status on CERAD neuropathological scores.
eTable 6. Adjusted pairwise comparisons from ordinal logistic regression models of stratified Sex–Race–AFR and APOEε4–Race–AFR subgroups predicting CERAD neuropathological score
eFigure 3. Associations of Sex–Race–AFR and APOEε4–Race–AFR Subgroups with CERAD neuropathological scores.
eTable 7. Neuropathological changes stratified by sex (n=2,268)
eTable 8. Association between amyloid pathology and cognitive abilities evaluated using Clinica Dementia Rating Sum of Boxes (CDR-SB) considering Sex and Amyloid interactions (n=2,268)
eTable 9. Association of sex and amyloid pathology with cognitive abilities (CDR-SB) considering interactions between sex and amyloid (n=2,268)
eTable 10. Association of sex and amyloid pathology with cognitive abilities (CDR-Global) considering interactions between sex and amyloid
eTable 11. Sensitivity analysis of the association between sex and amyloid deposition evaluated using the CERAD Neuropathological score across participants matched by functional cognitive level
eReferences

This supplemental material has been provided by the authors to give readers additional information about their work.

© 2026 Abu Raya M et al. *JAMA Neurology*.

eMethods

eFigure 1. Flowchart of the Study Participants

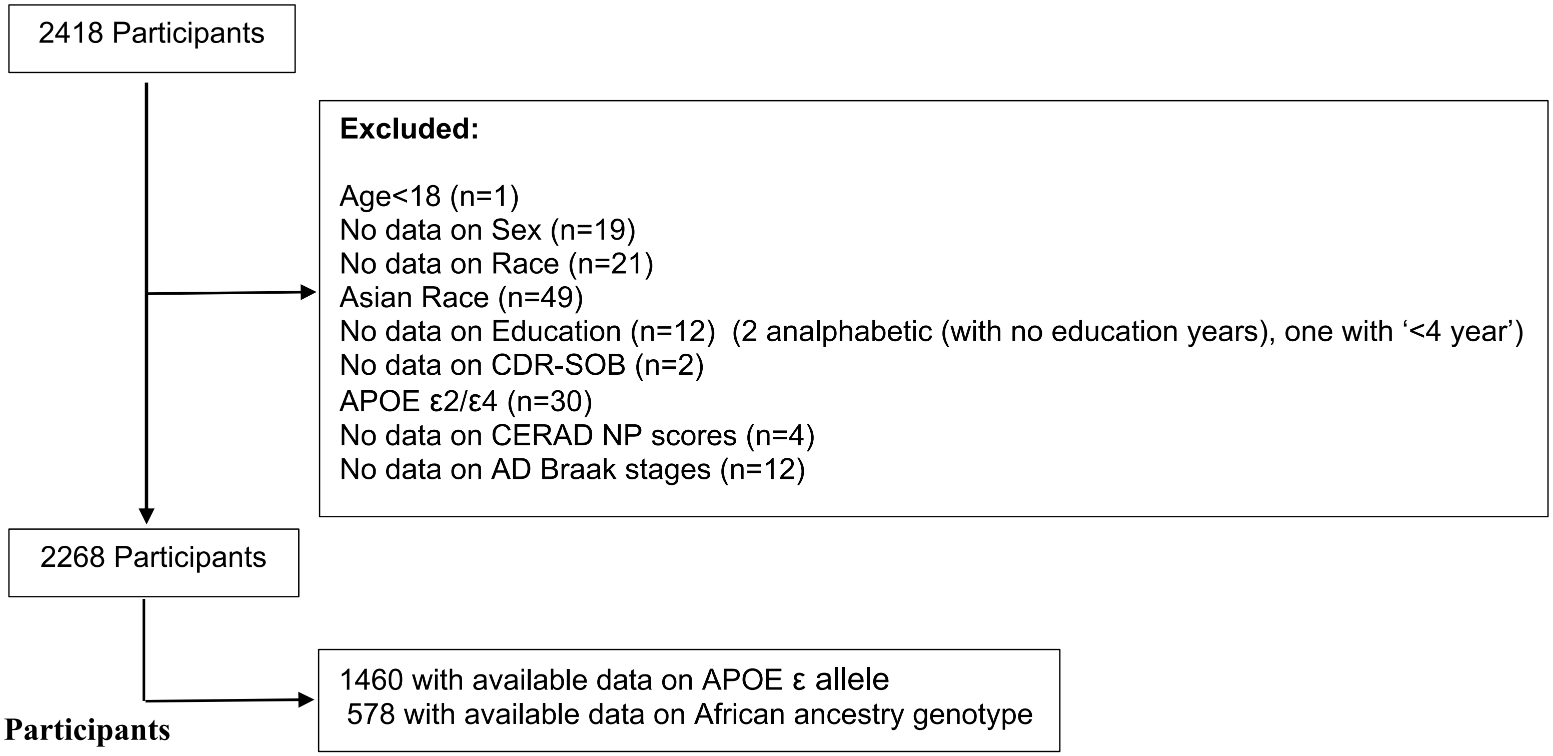

© 2026 Abu Raya M et al. *JAMA Neurology*.

Demographic and clinical variables

Sociodemographic information, including age, sex, education, and race, was collected from the next of kin (NOK). The NOK provided details on the deceased's education level and racial identity, categorized according to the Brazilian Institute of Geography and Statistics (IBGE) into Asian, Black, Indigenous, Pardo or White. Pardo defined as individuals who identify as mixed race, including combinations of White and Indigenous, White and Black, Black and Indigenous, or Black and another race.[1] For the analysis, self-declared Race was categorized as White or Black. Pardo and Black combined due to shared experiences of racism and health disparities,[2] consistent with prior Brazilian research. Asians were excluded due to small sample size (n=49). See **eFigure 1** for details on excluded data.

Neuropathology

Brain tissue processing followed standardized BAS protocols.[3–5] Brain tissue was collected within 24 hours after death. The **left hemisphere** was immersion-fixed in 4% paraformaldehyde, while selected regions from the **right hemisphere** were snap-frozen at −80 °C. Paraffin-embedded samples were obtained from the following anatomical regions: middle and inferior frontal gyri, superior and middle temporal gyri, angular gyrus, superior frontal and anterior cingulate cortices, primary visual cortex, anterior hippocampus, hippocampal formation at the lateral geniculate level, amygdala, basal ganglia (at the anterior commissure), thalamus, midbrain, pons, medulla, and cerebellum. Sections (5 µm thick) were stained with hematoxylin and eosin (H&E) for general histopathological evaluation. Immunohistochemistry was performed using the following antibodies: β-amyloid (4G8, 1:10,000; BioLegend #800701), phosphorylated tau (AT8, 1:400; Invitrogen MN1020), TDP-43 (1:500; BioLegend #829901), and α-synuclein (1:500; BioLegend #829901-BL), applied to selected sections based on clinical and pathological indications.[4,5]

© 2026 Abu Raya M et al. *JAMA Neurology*.

Lewy body disease (LBD) was classified using Braak staging for Parkinson's disease grouped in 3 categories: 0 to II, III to IV, and V to VI. LBP diagnosis was given for Braak PD stage ≥ III.[6] **Cerebrovascular pathology** was evaluated macroscopically during brain dissection and microscopically on paraffin sections. **Infarcts** were recorded based on size, number, and location, and considered present if ≥1 large infarct (≥1 cm), ≥3 lacunar infarcts (<1 cm) across three cortical regions, or infarcts in a strategic region (e.g., thalamus, caudate, basal forebrain, medial temporal lobe, or angular gyrus) were observed. **Small vessel disease (SVD)** included arteriolosclerosis and lipohyalinosis and was scored for extent and severity. SVD was considered present if moderate to severe changes were found in at least three cortical areas. **Cerebral amyloid angiopathy (CAA)** was defined by amyloid deposition in meningeal, cortical gray, and/or white matter vessels, and considered present when involvement was noted in at least three cortical regions. Less common pathologies (e.g., progressive supranuclear palsy, corticobasal degeneration, frontotemporal dementia, and **limbic-predominant age-related TDP-43 encephalopathy [LATE]**) were also recorded. **Hippocampal sclerosis** was defined by severe neuronal loss and gliosis in CA1 and subiculum. **TDP-43 pathology** was evaluated in the hippocampus, amygdala, and middle frontal gyrus.

Similar to previous studies from this group,[3–5,7] models included neuropathologies as binary variables (absent or present), Lewy body pathology was considered present when the Braak PD stage was III or higher. Infarcts were deemed present if there was at least one large infarct (≥1 cm), three or more lacunar infarcts (<1 cm) in three or more cortical areas, or infarcts in at least one strategic region such as the thalamus, basal forebrain, or medial temporal lobe. Small vessel disease was classified as present when moderate to severe vascular changes were identified in at least three cortical regions. Cerebral amyloid angiopathy was recorded as present if detected in three or more cortical areas. Vascular dementia was diagnosed when any of the criteria for infarcts, small vessel disease, or cerebral amyloid angiopathy were met. Mixed dementia was defined as the co-occurrence of Alzheimer's disease with either vascular dementia or Lewy body disease pathology.

Genetic testing

© 2026 Abu Raya M et al. *JAMA Neurology*.

APOE genotyping was carried out using real-time PCR targeting the rs429358 and rs7412 single-nucleotide polymorphisms that define the ε2/ε3/ε4 alleles[8]. Genotypes were grouped as APOEε4 carriers (≥1 ε4 allele: ε2/ε4, ε3/ε4, ε4/ε4) and non-carriers (ε2/ε2, ε2/ε3, ε3/ε3); ε2/ε4 cases (n=30) were excluded from APOEε4-stratified analyses because of the opposing biological effects of ε2 and ε4. Global tri-hybrid continental ancestry (European, African, Native American), available in 578 cases, was estimated using 47 ancestry-informative markers (AIMs) and the STRUCTURE 2.3.4 software[9]. The AIM panel and reference allele frequencies were derived from HapMap, the Human Genome Diversity Project (HGDP), and the New York Cancer Project (NYCP), as previously described[10,11]. Individuals were characterized both by their continuous African ancestry proportion (0–1) and by a dichotomous indicator using a 2% African ancestry threshold[10,11]. This cutoff approximates the upper bound of African ancestry observed in ~90% of individuals classified as White in reference Caucasian samples from the Human Genome Diversity and HapMap Projects and has been used in prior work in this cohort to distinguish minimal from appreciable African admixture.

Clinical data

Medical history and lifestyle factors, including diabetes, hypertension, dyslipidemia, smoking, heart failure, coronary artery disease, and stroke, were recorded using a semi-structured questionnaire and coded as binary variables (yes/no). BMI was calculated as weight (kg) divided by height squared (m^2) from supine measurements obtained before autopsy.

© 2026 Abu Raya M et al. *JAMA Neurology*.

Statistical analysis

Demographic, clinical, and pathological variables were summarized using means and standard deviations or frequencies and percentages, and compared by sex using t tests, χ^2 tests, or non-parametric tests as appropriate.

Associations between sex and amyloid pathology were evaluated using ordinal logistic regression models with CERAD neuritic plaque burden and Thal amyloid phase as outcomes. For each analysis, five nested models were fitted: Model 1 (unadjusted), Model 2 (demographics: age, race, education), Model 3 (adds APOEε4 status), Model 4 (adds vascular risk factors), and Model 5 (adds AD Braak stage to account for heterogeneity in overall neurofibrillary tangle burden at death, thereby isolating sex differences in amyloid deposition not explained solely by disease severity). Odds ratios (ORs) and 95% Wald confidence intervals (CIs) were calculated with males as the reference group. Analyses were repeated in a subset of individuals with genetic ancestry data (n=578), replacing self-reported race with African ancestry (AFR; ≥2% vs <2%).

Sensitivity analyses for CERAD and Thal outcomes included: (i) Braak stage–stratified models (**eTable 1)** to examine sex differences in amyloid pathology independent of tau; ii) models adjusting simultaneously for race and AFR to account for their distinct but overlapping contributions to sex differences in neuritic plaque burden; and (iii) models treating AFR as a continuous variable (0–1) to assess threshold versus gradient effects. Additional analyses were restricted to cognitively impaired individuals (CDR-Global ≥0.5) and to those with AD Thal phase ≥1, CERAD neuropathological scores ≥1, and AD Braak stage ≥1. In further sensitivity analyses, we fit multinomial logistic regression models treating CERAD neuropathological scores as a categorical outcome (**eTables 2–4).**

Interactions and joint effects

To assess whether sex–amyloid associations were modified by race/AFR and/or APOEε4, we used two complementary approaches. First, we extended Model 5 by adding two- and three-way interaction terms (e.g., Sex×APOEε4, Sex×Race×AFR). Second, we modeled a single categorical variable representing all combinations of sex with race/AFR, APOEε4, or both. Omnibus tests evaluated the overall effects of these interaction

© 2026 Abu Raya M et al. *JAMA Neurology*.

structures, and post hoc pairwise comparisons are reported in eTables 5–6. ORs from these models are reported using sum-to-zero contrasts relative to the grand mean.

Tau pathology at comparable amyloid levels

To assess whether tau pathology differs between women and men with similar levels of amyloid burden, participants were grouped into low (CERAD none/sparse) and high (CERAD moderate/frequent) amyloid categories. A multinomial logistic regression model predicted Braak stages (0–II, III–IV, V–VI) from Sex–Amyloid group, adjusting for age, race, and APOEε4 status. Post hoc pairwise comparisons of predicted probabilities were Bonferroni-corrected. In a complementary set of Braak stage–stratified analyses, participants were grouped into Braak stages 0–II, III–IV, and V–VI, and ordinal logistic regression was used to estimate associations between sex and (i) CERAD neuropathological scores and (ii) Thal phases within each stratum. Models were sequentially adjusted for demographics, APOEε4 status, and cardiovascular risk factors. Analyses were conducted within Braak strata to avoid adjusting for tau within the models, providing a non-causal sensitivity analysis of sex differences in amyloid burden independent of tau. Bootstrap 95% CIs (1,000 resamples) were used for inference **(eTable 1**).

Sensitivity Analyses

CERAD neuropathological scores as an outcome. In ordinal logistic regression models, sensitivity analyses included models adjusting for both race and AFR simultaneously to account for the distinct but overlapping contributions of genetic ancestry and social constructs of race that may confound with sex differences in neuritic plaque burden. Alternatively, AFR was modeled as a continuous variable to assess threshold versus gradient effects of ancestry on observed associations. We also conducted subgroup analyses restricted to individuals with cognitive impairment (defined as

© 2026 Abu Raya M et al. *JAMA Neurology*.

CDR-Global score≥ 0.5), and AD Thal phase, CERAD neuropathological scores and AD-Braak stage ≥1. Additionally, we fit multinomial logistic regression models treating CERAD neuropathological scores as a categorical outcome.

Cognition as an outcome

Linear regression models evaluated the associations of sex and amyloid pathology with CDR-SB. Primary predictors were sex, CERAD, and their interaction, with sequential adjustment: Model 1 (sex, CERAD, interaction), Model 2 (adds age, race, education, APOEε4), Model 3 (adds vascular risk factors), Model 4 (adds AD Braak stage), and Model 5 (adds co-pathologies: cerebral amyloid angiopathy, infarcts, arteriosclerosis, hippocampal sclerosis, PD Braak stage, LATE). Three-way interactions involving sex, race/AFR, and pathology were tested. Sensitivity analyses dichotomized CERAD (low vs high Aβ) and modeled CDR-Global as an ordinal outcome (eTables 87–109).

To evaluate the robustness of our main findings for cognition, we conducted two additional sensitivity analyses. First, we fit linear regression models with CDR-SB as a continuous outcome, treating CERAD neuropathological scores as a categorical predictor dichotomized as Low Aβ (none/sparse) versus High Aβ (moderate/frequent). Although the distribution of CDR-SB was non-normal, linear modeling was considered appropriate given the large sample size and consistency of findings across models. Second, we used a cumulative link model (ordinal logistic regression) to examine the association between amyloid pathology and CDR-Global scores, treated as an ordinal outcome with five levels (0.0, 0.5, 1.0, 2.0, 3.0). The interaction between sex and CERAD (high vs low Aβ) was included as a primary term of interest. Covariates included age, race, education, APOEε4 status, cardiovascular risk factors, AD Braak stage, and other co-pathologies (CAA, Braak-PD staging, LBD, infarcts, hippocampal sclerosis, TDP-

© 2026 Abu Raya M et al. *JAMA Neurology*.

43, and arteriosclerosis). Confidence intervals were estimated using 1,000 bootstrap resamples, and the proportional odds assumption was evaluated with the test of parallel lines, which showed no violations across predictors (all $p>0.05$). This approach allowed us to confirm that observed sex-by-amyloid interactions were robust across both continuous and ordinal cognitive outcomes and independent of key confounders.

To examine whether females exhibit greater amyloid pathology at comparable levels of cognitive function, we conducted a sensitivity analysis stratified by global cognitive status. Participants were grouped according to CDR-Global scores (0, 0.5, 1, 2, and 3). Within each stratum, ordinal logistic regression models estimated associations between sex and CERAD neuropathological scores, sequentially adjusted for demographics, APOEε4 status, and cardiovascular risk factors. Bootstrap 95% CIs (1,000 resamples) were used for inference. Some subgroups (e.g., CDR 0.5–1) were underpowered and results should be interpreted cautiously.

Model diagnostics

Model diagnostics were conducted for all linear and generalized linear models, including linear regression, ordinal regression, and multinomial logistic regression. For linear models, fit was assessed using F-tests, R^2, AIC/BIC, and root mean square error (RMSE). Assumptions of residual independence were evaluated using the Durbin–Watson test, and multicollinearity was assessed using variance inflation factors (VIFs); no violations were detected.

For multinomial logistic and ordinal regression models, model fit was evaluated using deviance statistics, AIC/BIC, pseudo-R^2, and likelihood ratio tests. Multicollinearity diagnostics indicated no violations. For all generalized linear models, 95% CIs were estimated using 1,000 bias-corrected and accelerated (BCa) bootstrap resamples. The proportional odds assumption in ordinal regression was evaluated using the test of parallel lines and was not violated.

© 2026 Abu Raya M et al. *JAMA Neurology*.

Given the non-normal distribution of CDR-SB, models that used CDR-SB as an outcome employed heteroscedasticity-consistent robust standard errors (HC3), together with 1,000 BCa bootstrap samples to estimate 95% CIs. These approaches were considered appropriate due to the large sample size and stable parameter estimates.

All analyses were conducted using jamovi (version 2.3, The jamovi Project) [12]and Python 3.9[13]. A two-sided alpha of 0.05 was used to define statistical significance. Model diagnostics, collinearity checks, and fit statistics are described in detail in the eResults.

© 2026 Abu Raya M et al. *JAMA Neurology*.

eResults

eTable 1. Sensitivity analyses examined the association between sex and amyloid deposition, using CERAD Neuropathological scores and Thal phase measures, stratified by Braak stage subgroups.

	**Female Sex***							
	**Model 1**		**Model 2**		**Model 3**		**Model 4**	
	**N**		**N**		**N**		**N**	
	**OR (95%CI)**	**p**	**OR (95%CI)**	**p**	**OR (95%CI)**	**p**	**OR (95%CI)**	**p**
**CERAD NP score (0-3) (Ordinal Outcome)**								
**Braak 0-II**	**1238**		**1238**		**740**		**731**	
	**1.25 (0.97- 1.66)**	**0.12**	**1.03 (0.78- 1.41)**	**0.85**	**1.00 (0.68- 1.51)**	**0.98**	**1.00 (0.68- 1.54)**	**0.99**
**Braak III-IV**	**725**		**725**		**496**		**493**	
	**1.28 (1.00- 1.69)**	**0.07**	**1.09 (0.84- 1.48)**	**0.55**	**1.19 (0.87- 1.77)**	**0.32**	**1.13 (0.78- 1.65)**	**0.53**
**Braak V-VI**	**305**		**305**		**224**		**219**	
	**1.85 (1.18- 3.32)**	**0.02**	**1.95 (1.19- 3.76)**	**0.01**	**1.88 (1.08- 4.29)**	**0.05**	**2.53 (1.35- 9.11)**	**0.009**
**Thal phase (0-5) (Ordinal Outcome)**								
**Braak 0-II**	**1068**		**1068**		**649**		**642**	
	**1.22 (0.96- 1.55)**	**0.11**	**1.06 (0.81- 1.39)**	**0.66**	**1.10 (0.79- 1.60)**	**0.57**	**1.06 (0.78- 1.61)**	**0.77**
**Braak III-IV**	**658**		**658**		**459**		**456**	
	**1.11 (0.84- 1.46)**	**0.47**	**0.97 (0.75- 1.36)**	**0.83**	**1.17 (0.83- 1.65)**	**0.38**	**1.21 (0.85- 1.79)**	**0.30**
**Braak V-VI**	**276**		**276**		**203**		**198**	
	**2.08 (1.35- 3.82)**	**0.005**	**2.24 (1.44- 4.60)**	**0.004**	**2.28 (1.23- 6.27)**	**0.02**	**2.49 (1.39- 7.26)**	**0.009**

Results from ordinal logistic regression analyses assessing the association between sex and (i) CERAD NP scores (None → Sparse → Moderate → Frequent) and (ii) Thal phase (Phase 0-5) as an outcome.
Analyses were conducted by Braak stage subgroup: (A) stages 0–II, (B) stages III–IV, and (C) stages V–VI. Odds ratios (ORs) for sex from the ordinal regression analysis are presented, with 95% confidence intervals (CIs) estimated from 1,000 bootstrap samples and corresponding P-values.

Models
Model 1: Unadjusted ordinal logistic regression model
Model 2: Ordinal logistic regression model adjusted for age, education, and reported race.
Model 3: Ordinal logistic regression model adjusted for age, education, race, and APOEε4.
Model 4: Ordinal logistic regression model adjusted for age, education, race, APOEε4, and † cardiovascular risk factors.
*Male is used as the reference group
Abbreviations: N is the number of cases; APOE ε4, ε4 allele of *apolipoprotein E gene; CERAD NP (Neuropathological score)*
† Cardiovascular risk factors were defined as the presence (yes/no) of hypertension, diabetes, dyslipidemia, coronary artery disease, heart failure, stroke, or smoking. Body mass index (BMI) was included as a continuous variable.

eTable 2. Association between sex and amyloid deposition evaluated using the CERAD neuropathological scores and Thal Phase staging in individuals with ancestry data (n=578)

	Female Sex*									
	Model 1		Model 2		Model 3		Model 4		Model 5	
	N		N		N		N		N	
	OR (95%CI)	p	OR (95%CI)	p	OR (95%CI)	p	OR (95%CI)	p	OR (95%CI)	p
**CERAD NP score (0-3) (Ordinal Outcome)**										
**A. Models adjusted for AFR**	578<br>2.00 (1.50- 2.88)	<.001	578<br>1.45 (1.09- 2.13)	0.03	578<br>1.67 (1.23- 2.52)	0.005	568<br>1.79 (1.29- 2.80)	0.003	568<br>1.45 (0.98- 2.47)	0.08
**B. Models Adjusted for AFR and Race**	578<br>2.00 (1.50- 2.88)	<.001	578<br>1.47 (1.05- 2.08)	0.030	578<br>1.70 (1.25- 2.55)	0.004	568<br>1.84 (1.29- 2.96)	0.002	568<br>1.50 (1.02- 2.53)	0.06
**Thal phase (0-5) (Ordinal Outcome)**										
**A. Models adjusted for AFR**	578<br>2.00 (1.46- 2.98)	<.001	447<br>1.55 (1.13- 2.25)	0.02	447<br>1.85 (1.30- 2.81)	0.001	439<br>1.98 (1.40- 3.25)	0.001	439<br>1.64 (1.06- 2.82)	0.02
**B. Models Adjusted for AFR and Race**	578<br>2.00 (1.46- 2.98)	<.001	447<br>1.55 (1.11- 2.25)	0.019	447<br>1.87 (1.35- 2.81)	0.001	439<br>2.01 (1.42- 3.37)	<.001	439<br>1.66 (1.08- 2.88)	0.02

© 2026 Abu Raya M et al. *JAMA Neurology*.

Results of separate ordinal logistic regression models predicting (i) CERAD neuropathological score and (ii) Thal amyloid phase from sex.
Panel A: adjusted for continuous African ancestry (AFR), **Panel B:** further adjusted for self-reported race. Odds ratios (ORs) for sex are presented with 95% confidence intervals estimated from 1,000 bootstrap samples and corresponding p-values.

Models
Model 1: Unadjusted ordinal logistic regression model
Model 2: Ordinal logistic regression model adjusted for **age, education, and race or/and African ancestry.**
Model 3: Ordinal logistic regression model adjusted for age, education, race or/and African ancestry**, and APOEε4.**
Model 4: Ordinal logistic regression model adjusted for age, education, race or/and African ancestry**,** APOEε4, and † **cardiovascular risk factors.**
Model 5: Ordinal logistic regression model adjusted for age, education, APOE e4, race or/and African ancestry**,** APOEε4, † cardiovascular risk factors, **and AD Braak stages.**
Abbreviations: N is the number of cases included in each model; APOE4, ε4 allele of apolipoprotein E gene; AFR, African Ancestry
*Male is used as the reference group
† Cardiovascular risk factors (yes/no): Hypertension, diabetes, dyslipidemia, coronary artery disease, cardiac failure, stroke, smoking, BMI

eTable 3. Association between sex and CERAD neuropathological score and Thal Phase in participants with Alzheimer's disease (AD) and cognitive impairment (n=447)

	Female Sex*									
	Model 1		Model 2		Model 3		Model 4		Model 5	
	N		N		N		N		N	
	OR (95%CI)	p	OR (95%CI)	p	OR (95%CI)	p	OR (95%CI)	p	OR (95%CI)	p
**CERAD score (0-3) (Ordinal Outcome)**										
**C. Models adjusted for Race**	447		447		302		295		295	
	1.86 (1.18- 2.94)	0.008	1.91 (1.19- 3.07)	0.007	1.94 (1.20- 3.13)	0.007	1.76 (1.06- 2.92)	0.03	0.94 (0.52- 1.68)	0.83
**D. Models Adjusted for AFR (+/-)**	112		112		112		108		108	
	2.35 (1.11- 5.03)	0.03	2.75 (1.25- 6.16)	0.01	2.88 (1.30- 6.53)	0.01	2.44 (1.03- 5.88)	0.04	1.55 (0.52- 4.62)	0.43
**Thal phase (0-5) (Ordinal Outcome)**										
**C. Models adjusted for Race**	387		387		267		260		260	
	1.84 (1.14- 2.98)	0.01	1.92 (1.16- 3.19)	0.011	1.90 (1.15- 3.15)	0.01	1.90 (1.11- 3.24)	0.019	1.04 (0.59- 1.82)	0.90
**D. Models Adjusted for AFR (+/-)**	85		85		85		81		81	
	2.06 (0.87- 4.94)	0.10	2.15 (0.83- 5.66)	0.12	2.43 (0.92- 6.54)	0.07	3.05 (1.04- 9.16)	**0.04**	1.62 (0.50- 5.34)	0.42

Results from ordinal logistic regression analyses assessing the association between sex and CERAD neuropathological scores and Thal phase as an outcome in **a subgroup with clinical impairment (**defined as CDR-Global score≥ 0.5), and AD Thal phase, CERAD neuropathological scores and AD- Braak stage ≥1 . Analysis (**A)** included models adjusted for Race, and **(B)** for African ancestry (AFR)(dichotomized variable +/-). Odds ratios (ORs) for sex from the ordinal regression analysis are presented, with 95% confidence intervals (CIs) and p-values.
Models
Model 1: Unadjusted ordinal logistic regression model
Model 2: Ordinal logistic regression model adjusted for **age, education, and race or African ancestry.**
Model 3: Ordinal logistic regression model adjusted for age, education, race or African ancestry**, and APOEε4.**

© 2026 Abu Raya M et al. *JAMA Neurology*.

Model 4: Ordinal logistic regression model adjusted for age, education, race or African ancestry**,** APOEε4, and † **cardiovascular risk factors.**
Model 5: Ordinal logistic regression model adjusted for age, education, race or African ancestry**,** APOEε4, † cardiovascular risk factors, **and AD Braak stages.**
Abbreviations: N is the number of cases included in each model; APOE4, ε4 allele of apolipoprotein E gene
*Male is used as the reference group
† Cardiovascular risk factors (yes/no): Hypertension, diabetes, dyslipidemia, coronary artery disease, cardiac failure, stroke, smoking, BMI (body mass index) was included as a continuous variable.

© 2026 Abu Raya M et al. *JAMA Neurology*.

eTable 4. Association between sex and neuropathologic outcomes, CERAD neuropathological score as a categorical variable

	Sex (Female-Male)*									
Outcome: CERAD NP score	Model 1		Model 2		Model 3		Model 4		Model 5	
	N		N		N		N		N	
	OR (95%CI)	p	OR (95%CI)	p	OR (95%CI)	p	OR (95%CI)	p	OR (95%CI)	p
**A. Models adjusted for Race**										
**Moderate - None or Sparse**	**2268**		**2268**		**1460**		**1443**		**1443**	
	1.84 (1.37-2.49)	**<.001**	1.46 (1.06-2.00)	**0.02**	1.50 (1.09-2.08)	**0.01**	1.48 (1.05-2.08)	**0.023**	1.28 (0.89-1.84)	0.18
**Frequent – None or Sparse**	2.82 (2.01-4.00)	**<.001**	2.10 (1.46-3.01)	**<.001**	2.18 (1.50-3.17)	**<.001**	2.14 (1.45-3.18)	**<.001**	1.25 (0.74-2.13)	0.407
**B. Models Adjusted for AFR**										
**Moderate - None or Sparse**	**578**		**578**		**578**		**568**		**568**	
	1.89 (1.19-2.99)	**0.007**	1.38 (0.83-2.27)	0.21	1.50 (0.89-2.51)	0.13	1.55 (0.89-2.69)	0.121	1.51 (0.84-2.70)	0.17
**Frequent – None or Sparse**	3.13 (1.76-5.57)	**<.001**	2.31 (1.25-4.27)	**0.008**	2.64 (1.39-5.02)	**0.003**	2.59 (1.31- 5.10)	**0.006**	2.78 (1.01-7.66)	**0.05**

Reference: Male sex

Results from multinomial logistic regression analyses assessing the association between sex and CERAD neuropathological scores as a categorical variable. Analysis (**A**) included models adjusted for Race, and **(B)** for African ancestry (AFR). Odds ratios (ORs) for sex from the ordinal regression analysis are presented, with 95% confidence intervals (CIs) and p-values.

Models

Model 1: Unadjusted multinomial logistic regression model

Model 2: Multinomial logistic regression model adjusted for **age, education, and reported race (block A) or African ancestry (block B)**

Model 3: Multinomial logistic regression model adjusted for age, education, race or African ancestry**, and APOEε4.**

Model 4: Multinomial logistic regression model adjusted for age, education, race or African ancestry**,** APOEε4, and † **cardiovascular risk factors.**

Model 5: Multinomial logistic regression model adjusted for age, education, race or African ancestry**,** APOEε4, †cardiovascular risk factors, **and AD Braak stages.**

Abbreviations: N is the number of cases included in each model; APOEε4, ε4 allele of apolipoprotein E gene

† Cardiovascular risk factors (yes/no): hypertension, diabetes, dyslipidemia, coronary artery disease, cardiac failure, stroke, smoking. BMI (body mass index) was included as a continuous variable.

© 2026 Abu Raya M et al. *JAMA Neurology*.

eTable 5. Summary of omnibus chi-square statistics (χ^2) and post hoc comparisons from ordinal logistic regression models testing Sex–Race–APOEε4 interactions and stratified subgroup models predicting CERAD neuropathological score

Interaction terms	Omnibus χ^2, p-value	Significant post-hoc comparisons	OR (95% CI)	SE	P Bonferroni	Stratified grouping	Omnibus χ^2, p-value	Significant post-hoc comparisons	OR (95% CI)	SE	P Bonferroni
**Model A. adjusted for race**											
Sex*Race	χ^2 =103, p=<.001	M-W vs. M-B	0.66 (0.52–0.84)	0.13	0.17	Sex-Race	χ^2 =446, p=<.001	M-B vs. M-W	0.63 (0.50, 0.80)	0.12	0.08
		F-W vs. M-B	1.74 (0.90-3.36)	0.34	0.03			M-B vs. F-W	0.53 (0.39, 0.72)	0.10	0.005
		F-W vs. F-B	0.66 (0.52–0.84)	0.12	0.15			F-B vs. F-W	0.62 (0.46, 0.85)	0.11	0.05
Sex*APOEε4	χ^2 =44, p=<.001	M-C vs. M-NC	4.17 (0.91–19.1)	0.78	<.001	Sex-APOEε4	χ^2 =530, p=<.001	M-C vs. M-NC	3.72 (1.01–13.72)	0.67	< .001
		F-C vs. F-NC	3.82 (0.97–15.11)	0.70	<.001			F-C vs. F-NC	3.45 (1.05–11.36)	0.61	< .001
		M-C vs. F-NC	3.45 (3.13-3.85)	0.06	<.001			M-C vs. F-NC	3.08 (1.06–8.96)	0.55	< .001
		F-C vs. M-NC	4.57 (0.82–25.49)	0.88	<.001			F-C vs. M-NC	4.17 (0.94–18.50)	0.76	< .001
Race*APOEε4	χ^2 =349, p=<.001	W-C vs. W-NC	2.88 (1.2–6.93)	0.45	<.001	Race-APOEε4	χ^2 =361, p=<.001	W-C vs. W-NC	2.88 (2.00, 4.14)	0.45	<.001
		B-C vs. B-NC	5.53 (0.51–60.16)	1.22	<.001			B-C vs. B-NC	5.52 (3.87, 7.88)	1.22	<.001
		B-NC vs. W-NC	0.48 (0.4–0.56)	0.08	<.001			B-NC vs. W-NC	0.48 (0.34, 0.67)	0.08	<.001
		B-C vs. W-NC	2.63 (1.04–6.66)	0.47	<.001			B-C vs. W-NC	2.62 (1.79, 3.84)	0.47	<.001
		B-C vs. W-C	0.91 (0.64–1.31)	0.18	1			B-C vs. W-C	0.91 (0.63, 1.32)	0.18	1
		B-NC vs. W-C	0.16 (0.15–0.18)	0.03	<.001			W-C vs. B-NC	6.07 (4.24, 8.69)	1.22	<.001
Sex*Race*APOEε4	χ^2=0.01, p=0.899	M-W-C vs. M-W-NC	2.76 (0.85–8.97)	0.6	<.001	Sex-Race-APOEε4	χ^2 =580, p=<.001	M-W-C vs. M-W-NC	2.77 (0.85–8.97)	0.60	< .001
		M-B-C vs. M-B-NC	6.94 (0.09–532.73)	2.21	<.001			M-B-C vs. M-B-NC	6.94 (0.09–532.73)	2.22	< .001
		F-W-C vs. F-W-NC	3.01 (0.83–10.9)	0.66	<.001			F-W-C vs. F-W-NC	3.01 (0.83–10.9)	0.66	<.001
		F-B-C vs. F-B-NC	4.48 (0.32–63.35)	1.35	<.001			F-B-C vs. F-B-NC	4.48 (0.32–63.35)	1.35	<.001
		M-B-NC vs. M-W-NC	0.42 (0.34–0.52)	0.11	0.02			M-B-NC vs. M-W-NC	0.42 (0.34–0.52)	0.11	0.02
		M-B-NC vs. F-W-NC	0.37 (0.31–0.45)	0.09	0.003			M-B-NC vs. F-W-NC	0.37 (0.31–0.45)	0.09	0.003
		F-W-C vs. M-W-NC	3.4 (0.75–15.42)	0.77	<.001			F-W-C vs. M-W-NC	3.4 (0.75–15.42)	0.77	<.001
		F-B-C vs. M-B-NC	6.37 (0.13–310.97)	1.98	<.001			F-B-C vs. M-B-NC	6.37 (0.13–310.97)	1.98	<.001
		F-B-NC vs. M-W-C	0.22 (0.19–0.24)	0.06	<.001			F-B-NC vs. M-W-C	0.22 (0.19–0.24)	0.06	<.001
		F-B-NC vs. M-B-C	0.2 (0.18–0.23)	0.06	<.001			F-B-NC vs. M-B-C	0.2 (0.18–0.23)	0.06	<.001
		F-W-NC vs. M-W-C	0.41 (0.35–0.49)	0.09	<.001			F-W-NC vs. M-W-C	0.41 (0.35–0.49)	0.09	<.001
		F-B-NC vs. F-W-C	0.18 (0.16–0.19)	0.05	<.001			F-B-NC vs. F-W-C	0.18 (0.16–0.19)	0.05	<.001
		F-B-C vs. M-W-NC	2.69 (0.72–10.08)	0.67	0.002			F-B-C vs. M-W-NC	2.69 (0.72–10.08)	0.67	0.002
		M-W-C vs. F-W-NC	2.44 (0.88–6.77)	0.52	<.001			M-W-C vs. F-W-NC	2.44 (0.88–6.77)	0.52	<.001
		M-B-C vs. F-W-NC	2.59 (0.70–9.53)	0.67	0.006			M-B-C vs. F-W-NC	2.59 (0.70–9.53)	0.67	0.006
		F-B-C vs. F-W-NC	2.37 (0.76–7.39)	0.58	0.01			F-B-C vs. F-W-NC	2.37 (0.76–7.39)	0.58	0.01
		M-W-C vs. M-B-NC	6.55 (0.17–254.45)	1.87	<.001			M-W-C vs. M-B-NC	6.55 (0.17–254.45)	1.87	<.001
		F-W-C vs. M-B-NC	8.07 (0.08–827.22)	2.36	<.001			F-W-C vs. M-B-NC	8.07 (0.08–827.22)	2.36	<.001
		F-W-NC vs. M-B-NC	2.68 (0.71–10.16)	0.68	0.003			F-W-NC vs. M-B-NC	2.68 (0.71–10.16)	0.68	0.003
**Model B. adjusted for AFR**											
Sex*AFR	χ^2 =5, p=0.028	F-AFR- vs. M-AFR+	2.82 (0.43–18.35)	0.96	0.013	Sex-AFR	χ^2=193, p=<.001	M-AFR+ vs. F-AFR-	0.38 (0.31–0.48)	0.115	0.008
Sex*APOEε4	χ^2 =32, p=<.001	M-C vs. M-NC	3.29 (0.44–24.70)	1.03	0.001	Sex-APOEε4	χ^2=237, p=<.001	M-C vs. M-NC	3.31 (0.49–22.16)	0.97	0.001
		F-C vs. F-NC	4.99 (0.22–110.83)	1.58	0.001			F-C vs. F-NC	4.94 (0.32–76.36)	1.40	0.001
		F-C vs. M-NC	6.75 (0.06–815.54)	2.45	0.001			F-C vs. M-NC	6.36 (0.15–268.71)	1.91	0.001
		M-C vs. F-NC	2.44 (1.89-3.13)	0.13	0.02			M-C vs. F-NC	2.57 (0.63–10.54)	0.72	0.004
		F-C vs. M-C	2.05 (0.50–8.42)	0.72	0.25			F-C vs. M-C	1.92 (0.56–6.57)	0.63	0.28
		F-NC vs. M-NC	1.35 (0.69–2.67)	0.35	1.00			F-NC vs. M-NC	1.29 (0.71–2.35)	0.31	1.00
AFR *APOEε4	χ^2 =5, p=0.025	AFR-C vs. AFR-NC	4.00 (0.15–105.66)	1.67	0.005	AFR-APOEε4	χ^2 =234, p=<.001	AFR-C vs. AFR-NC	3.77 (0.18–76.96)	1.54	0.007

© 2026 Abu Raya M et al. *JAMA Neurology*.

		AFR+C vs. AFR+NC	4.11 (0.62–27.32)	0.97	0.001			AFR+C vs. AFR+NC	4.18 (0.61–28.68)	0.98	0.0001
		AFR+C vs. AFR-NC	2.39 (0.62–9.26)	0.69	0.02			AFR+C vs. AFR-NC	2.39 (0.63–9.01)	0.68	0.01
		AFR+NC vs. AFR-C	0.15 (0.13–0.16)	0.06	0.001			AFR-C vs. AFR+NC	6.60 (0.05–865.97)	2.49	0.001
		AFR+NC vs. AFR-NC	0.58 (0.43–0.78)	0.15	0.22			AFR+NC vs. AFR-NC	0.57 (0.43–0.76)	0.15	0.17
		AFR-C vs. AFR+C	0.60 (0.38–0.95)	0.24	1.00			AFR+C vs. AFR-C vs.	0.63 (0.39–1.03)	0.25	1.00
Sex*AFR* APOEε4	χ^2=0.03, *p*=0.852	M-C-AFR- vs. M-NC-AFR-	4.28 (0.04–495.76)	2.42	0.29	Sex-AFR-APOEε4	χ^2 =248, p=<.001	M-C-AFR- vs. M-NC-AFR-	4.28 (0.04–495.76)	2.42	0.29
		F-C-AFR- vs. F-NC-AFR-	3.54 (0.05–258.54)	2.19	1.00			F-C-AFR- vs. F-NC-AFR-	3.54 (0.05–258.54)	2.19	1.00
		M-C-AFR+ vs. M-NC-AFR+	3.01 (0.40–22.66)	1.03	0.04			M-C-AFR+ vs. M-NC-AFR+	3.01 (0.40–22.66)	1.03	0.04
		F-NC-AFR+ vs. F-C-AFR+	0.18 (0.01–5.65)	1.75	0.001			F-NC-AFR+ vs. F-C-AFR+	0.18 (0.01–5.65)	1.74	0.001
		M-NC-AFR- vs. F-C-AFR+	0.213 (0.004-12.5)	2.08	0.01			F-C-AFR+ vs. M-NC-AFR-	0.213 (0.0036-12.5)	2.08	0.01
		M-NC-AFR+ vs. M-C-AFR-	0.18 (0.001 –33.33)	2.73	0.01			M-C-AFR- vs. M-NC-AFR+	0.18 (0.15–0.21)	0.09	0.01
		F-C-AFR- vs M-NC-AFR+	8.44 (0.00–163735.61)	5.04	0.01			M-NC-AFR+ vs. F-C-AFR-	0.12 (0.10–0.14)	0.07	0.01
		M-NC-AFR+ vs. F-C-AFR+	0.16 (0.003-9.09)	2.06	0.001			M-NC-AFR+ vs. F-C-AFR+	0.16 (0.003-9.09)	2.06	0.001
		F-NC-AFR+ vs. M-C-AFR-	0.20 (0.17–0.24)	0.10	0.02			F-NC-AFR+ vs. M-C-AFR-	0.20 (0.17–0.24)	0.10	0.02
		F-NC-AFR+ vs. M-C-AFR+	0.37 (0.29–0.47)	0.12	0.08			M-C-AFR+ vs. F-NC-AFR+	2.69 (0.47–15.35)	0.89	0.08
		F-NC-AFR- vs. M-C-AFR-	0.42 (0.28–0.65)	0.22	1.00			M-C-AFR- vs. F-NC-AFR-	2.35 (0.22–25.06)	1.21	1.00
		F-C-AFR- vs. F-NC-AFR+	7.53 (0.00–45292.79)	4.44	0.02			F-NC-AFR+ vs.F-C-AFR-	0.13 (0.11–0.16)	0.08	0.02
		F-NC-AFR- vs M-C-AFR+	0.79 (0.44–1.43)	0.30	1.00			M-C-AFR+ vs. F-NC-AFR-	1.26 (0.49–3.27)	0.49	1.00

Omnibus chi-square statistics (χ^2) and p-values for interaction terms, along with post hoc comparisons from interaction models and stratified subgroup models predicting CERAD neuropathological score, are presented.

Model A adjusted for Race, and **Model B** adjusted for genetically defined African ancestry (AFR). For each interaction term, statistically significant pairwise comparisons (Bonferroni-adjusted $p < 0.05$) are reported alongside their odds ratios (ORs) and adjusted p-values. Corresponding results from models using stratified grouping variables (e.g., Sex-Race, Sex-APOEε4) are included for comparison.

Abbreviations: AFR, African ancestry; APOEε4, apolipoprotein E ε4 carrier status; F, Female; M, Male; W, White; B, Black; C, APOEε4 Carriers; NC, APOEε4 Non carriers.

© 2026 Abu Raya M et al. *JAMA Neurology*.

eFigure 2. *Three-way interactions of sex, race/ancestry, and APOEε4 status on* ***CERAD*** n***europathological scores.***
(A) Forest plot showing odds ratios (ORs) and 95% confidence intervals (CIs) for the association between sex, self-reported race (White or Black), and APOEε4 carriership (Carriers (C), Non carriers (NC)). The three-way interaction term (Sex × Race × APOEε4) was not statistically significant ($\chi^2 = 0.02$, $p = 0.90$). However, stratified estimates suggest that among non-carriers, Black individuals had significantly lower amyloid burden compared to White individuals. Among carriers, these differences were attenuated and all groups exhibited similarly elevated odds. (B) Forest plot showing odds ratios for the interaction between sex, genetically inferred African ancestry (AFR), and APOEε4 status. The three-way interaction (Sex × AFR × APOE4) was also not significant ($\chi^2 = 0.04$, $p = 0.85$). Descriptively, APOEε4 carrier females with low African ancestry (AFR-) had the highest odds of neuritic plaque pathology. Among non-carriers, individuals with AFR+ ancestry—particularly males—showed lower amyloid burden than their AFR– counterparts.

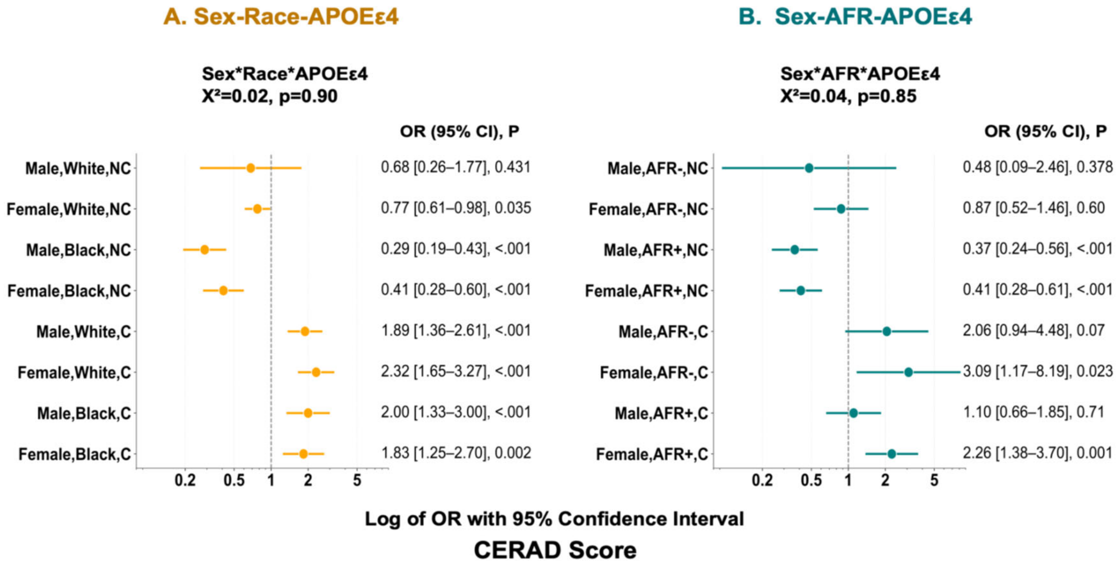

All models adjust for age and AD Braak stage. Dots represent ORs, and lines represent 95% CIs on a log scale. Asterisks indicate Bonferroni-significant pairwise comparisons ($p < 0.05$).
Abbreviations: APOE4, apolipoprotein E ε4; C, carrier; NC, non-carrier; AFR+, high African ancestry; AFR–, low African ancestry; M, male; F, female; OR, odds ratio.

© 2026 Abu Raya M et al. *JAMA Neurology*.

eTable 6. Adjusted pairwise comparisons from ordinal logistic regression models of stratified Sex–Race–AFR and APOEε4–Race–AFR subgroups predicting CERAD neuropathological score

Comparison			CERAD score			Panel B	Comparison		CERAD score		
**Sex,Race,AFR**	**vs**	**Sex,Race,AFR**	**OR (95%CI)**	**SE**	**$p_{bonferroni}$**	**Race,AFR,APOEε4**	**vs**	**Race,AFR,APOEε4**	**OR (95%CI)**	**SE**	**$p_{bonferroni}$**
Female,White,AFR-		Male,White,AFR-	1.76 [0.48–6.47]	0.663	1.00	White,AFR+,NC		White,AFR-,NC	0.77 [0.51–1.16]	0.210	1.00
Male,White,AFR+		Male,White,AFR-	0.82 [0.47–1.44]	0.285	1.00	Black,AFR+, NC		White,AFR-,NC	0.29 [0.24–0.35]	0.097	**0.004**
Female,White,AFR+		Male,White,AFR-	1.15 [0.53–2.50]	0.396	1.00	White, AFR-,C		White,AFR-,NC	3.88 [0.17–91.19]	1.610	**0.02†**
Male,Black,AFR+		Male,White,AFR-	0.40 [0.29–0.55]	0.162	0.35	White, AFR+,C		White,AFR-,NC	2.45 [0.47–12.83]	0.844	0.136
Female,Black,AFR+		Male,White,AFR-	0.68 [0.42–1.09]	0.243	1.00	Black,AFR+,C		White,AFR-,NC	2.21 [0.52–9.39]	0.739	0.27
Male,White,AFR+		Female,White,AFR-	0.47 [0.35–0.63]	0.151	0.28	Black,AFR+, NC		White,AFR+,NC	0.37 [0.30–0.47]	0.116	**0.02**
Female,White,AFR+		Female,White,AFR-	0.65 [0.44–0.97]	0.205	1.00	White, AFR-,C		White,AFR+,NC	5.05 [0.11–241.5]	1.973	**< .001†**
Male,Black,AFR+		Female,White,AFR-	0.23 [0.19–0.27]	0.088	**0.002**	White, AFR+,C		White,AFR+,NC	3.19 [0.45–22.85]	1.004	**0.003**
Female,Black,AFR+		Female,White,AFR-	0.38 [0.30–0.49]	0.130	**0.07**	Black,AFR+,C		White,AFR+,NC	2.87 [0.51–16.04]	0.878	**0.008**
Female,White,AFR+		Male,White,AFR+	1.40 [0.64–3.06]	0.401	1.00	White, AFR-,C		Black,AFR+, NC	13.57 [0.0–1878284.6]	6.040	**< .001†**
Male,Black,AFR+		Male,White,AFR+	0.48 [0.34–0.68]	0.173	0.63	White, AFR+,C		Black,AFR+, NC	8.58 [0.02–4725.25]	3.220	**< .001†**
Female,Black,AFR+		Male,White,AFR+	0.82 [0.50–1.34]	0.250	1.00	Black,AFR+,C		Black,AFR+, NC	7.72 [0.03–1988.08]	2.832	**< .001†**
Male,Black,AFR+		Female,White,AFR+	0.35 [0.27–0.44]	0.124	**0.05**	White, AFR+,C		White, AFR-,C	0.63 [0.37–1.09]	0.276	1.00
Female,Black,AFR+		Female,White,AFR+	0.59 [0.42–0.83]	0.177	1.00	Black,AFR+,C		White, AFR-,C	0.57 [0.35–0.92]	0.247	1.00
Female,Black,AFR+		Male,Black,AFR+	1.70 [0.50–5.77]	0.625	1.00	Black,AFR+,C		White, AFR+,C	0.9 [0.47–1.71]	0.327	1.00

Bonferroni-adjusted pairwise comparisons are shown from two ordinal logistic regression models evaluating CERAD neuropathological scores across stratified subgroups: (A) Race–AFR–APOEε4 (adjusted for age, sex, education, and Braak stage), and (B) Sex–Race–AFR (adjusted for age, education, APOEε4 status, and Braak stage).
Models used deviation (sum-to-zero) coding, with odds ratios (ORs) reflecting each group's deviation from the grand mean. The "Ratio" column denotes ORs comparing CERAD burden between specific subgroups, where values >1 indicate higher odds of greater plaque burden in the first group.
These models were constructed to evaluate how intersectional biological and social factors—including APOEε4 genotype, self-identified race, genetically defined African ancestry (AFR), and sex—contribute to differences in CERAD neuropathological scores. While three-way interaction terms were not statistically significant, these post hoc comparisons identify subgroup-level differences in CERAD neuropathological scores. Significant differences (Bonferroni-adjusted $p < 0.05$) are highlighted and correspond to findings in eFigure 3.
Abbreviations: OR, odds ratio; SE, standard error; APOEε4, apolipoprotein E ε4 carrier status; AFR, African genetic ancestry; C, carriers; NC, non-carriers; CERAD, Consortium to Establish a Registry for Alzheimer's Disease.
† Comparisons with low precision with large standard error (SE > 1.5) or wide CI range due to sparse data; interpret with caution.

© 2026 Abu Raya M et al. *JAMA Neurology*.

eFigure 3. Associations of Sex–Race–AFR and APOEε4–Race–AFR Subgroups with CERAD neuropathological scores. Forest plots show odds ratios (ORs) and 95% confidence intervals (CIs) from ordinal logistic regression models predicting CERAD neuritic plaque scores. **Panel A** includes subgroups defined by sex, reported race, and African genetic ancestry (AFR); **Panel B** includes APOEε4 carrier status, race, and AFR. Models adjust for age, education, Braak stage, and either sex or APOEε4 status, where applicable.

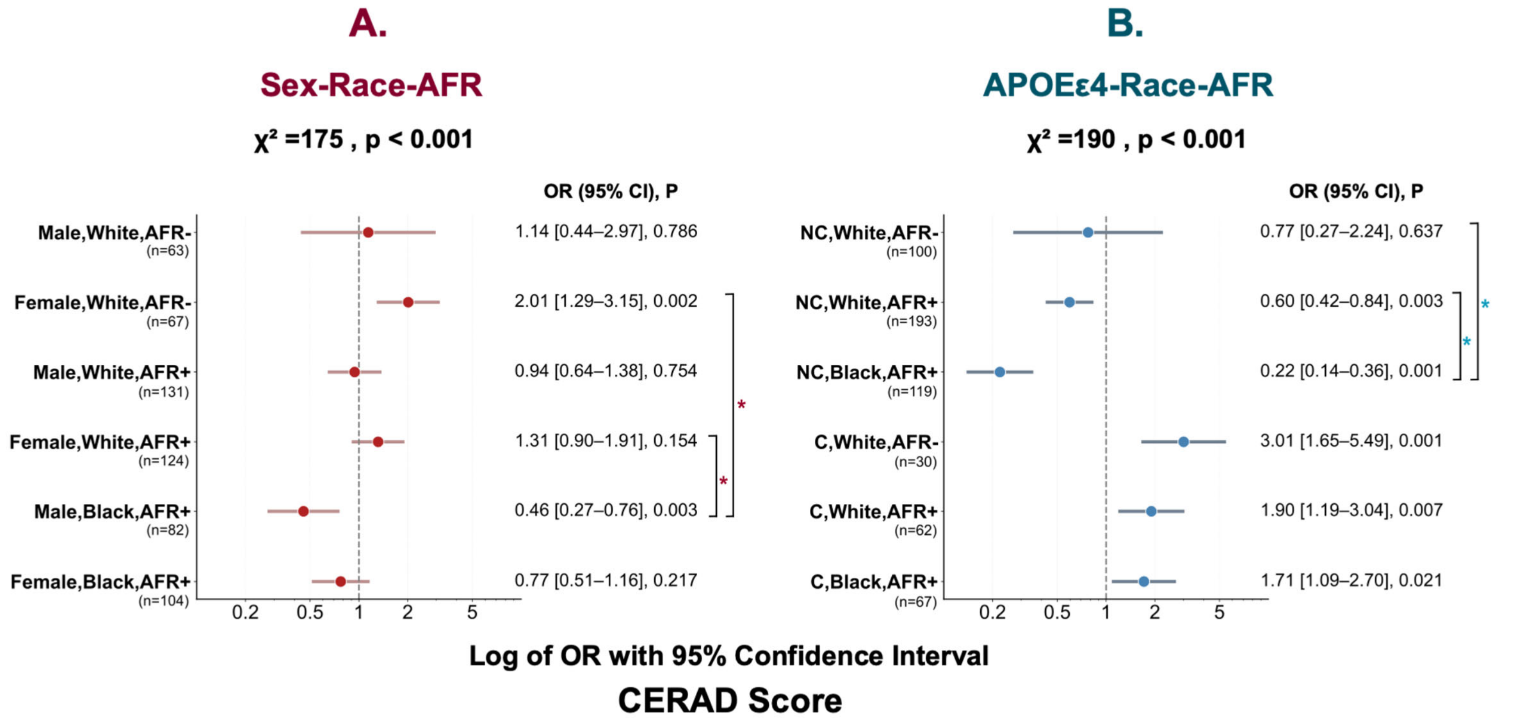

Estimates were derived using deviation (sum-to-zero) coding, where each OR reflects the subgroup's deviation from the grand mean. Asterisks indicate Bonferroni-adjusted pairwise comparisons with $p < 0.05$. Full post hoc comparisons are provided in e**Table 6**.

Abbreviations: OR odds ratio; CI, confidence interval; APOEε4, apolipoprotein E ε4 carrier status; NC, non-carrier; C, carrier; AFR, African genetic ancestry.

© 2026 Abu Raya M et al. *JAMA Neurology*.

eTable 7. Neuropathological changes stratified by sex (n=2,268)

Categorical variables are shown as counts (% of available data) and compared using Chi-squared tests.
*N is the number of non-missing value.
Abbreviations: PD, Parkinson's Disease; LBD, Lewy Body Disease; CAA, CERAD NP: CERAD **neuropathological score;** Cerebral Amyloid Angiopathy; TDP, TDP-43, Transactive response DNA binding protein of 43 kDa.

Characteristics, N (%)	*N	Total 2268	Male 1152 (51%)	Female 1116 (49%)	p
**Pathology Diagnosis**					
AD Thal Phase	2002		Male	Female	<0.001
0		933 (47%)	538 (52%)	395 (41%)	
1		484 (24%)	258 (25%)	226 (23%)	
2		237 (12%)	112 (11%)	125 (13%)	
3		348 (17%)	124 (12%)	224 (23%)	
CERAD NP score	2268				<0.001
None/Sparse		1696 (75%)	946 (82%)	750 (67%)	
Moderate		322 (14%)	128 (11%)	194 (17%)	
Frequent		250 (11%)	78 (7%)	172 (15%)	
AD Braak stage	2268				<0.001
0-II		1238 (55%)	712 (62%)	526 (47%)	
III-IV		725 (32%)	351 (31%)	374 (34%)	
V-VI		305 (13%)	89 (8%)	216 (19%)	
ABC composite score	2002				<.001
None		931 (47%)	538 (52%)	393 (41%)	
Low		556 (28%)	298 (29%)	258 (27%)	
Intermediate		221 (11%)	104 (10%)	117 (12%)	
High		294 (15%)	92 (9%)	202 (21%)	
PD Braak stage	2170				0.22
0-II		1989(92%)	1012/ (92%)	977/ (91%)	
III-IV		101 (5%)	53 (5%)	48 (5%)	
V-VI		80 (4%)	33 (3%)	47 (4%)	
CAA (present)	2254	317 (14%)	131 (11%)	186 (17%)	<0.001
LBD (present)	2191	258 (12%)	121 (11%)	137 (13%)	0.23
TDP-43 (present)	1867	187 (10%)	70 (7%)	117 (13%)	<0.001
Hippocampal Sclerosis (presence)	2253	77 (3%)	26 (2%)	51 (5%)	0.002
TDP_ LATE	1822				<.001
None		1681 (92%)	896 (95%)	785 (89%)	
Amygdala		56 (3%)	21 (2%)	35 (4%)	
Hippocampus		73 (4%)	22 (2%)	51 (6%)	
Medial Frontal Gyrus		12 (1%)	4 (1%)	8 (1%)	
Arteriosclerosis (presence)	2102	908 (43%)	444 (42%)	464 (44%)	0.33
Infarct (presence)	2092	221 (11%)	123 (12%)	98 (9%)	0.10

© 2026 Abu Raya M et al. *JAMA Neurology*.

eTable 8. Association between amyloid pathology and cognitive abilities evaluated using Clinica Dementia Rating Sum of Boxes (CDR-SB) considering Sex and Amyloid interactions (n=2,268)

Continuous Outcome: CDR-SB	Model 1		Model 2		Model 3		Model 4		Model 5	
	N=2268		N=1460		N=1443		N=1443		N=1443	
	β (95%CI)	p	β (95%CI)	p	β (95%CI)	p	β (95%CI)	p	β (95%CI)	p
**Sex**	0.20 (0.13-0.27)	0.07	0.10 (-0.002-0.18)	0.27	0.14 (0.05-0.23)	0.90	0.08 (-0.02-0.17)	0.63	0.08 (-0.02-0.18)	0.73
**CERAD NP score (Ordinal)**	0.41 (0.36-0.45)	**<.001**	0.35 (0.28-0.40)	**<.001**	0.32 (0.26- 0.38)	**<.001**	0.11 (0.04-0.18)	**0.004**	0.11 (0.04-0.20)	**0.009**
**Sex* CERAD Score**	0.17 (0.08-0.27)	**<.001**	0.20 (0.09-0.30)	**<.001**	0.18 (0.07-0.29)	**0.001**	0.08 (-0.02-0.18)	**0.13**	0.09 (-0.03-0.21)	0.17

Results from generalized linear models examining the association between AD CERAD scores and CDR-SB, including interaction terms with sex. Standardized estimates (β), 95% confidence intervals (CI), and p-values are shown. AD CERAD score was modeled as an ordinal variable (0–3) and treated as a continuous predictor. Models used HC3 robust standard errors and 1,000 bootstrap samples.

Models:

Model 1: sex +D CERAD neuropathological score+ Sex* CERAD neuropathological score
Model 2: Model 1 + age + race + education + APOEε4
Model 3: Model 2 + cardiovascular risk factors
Model 4: Model 3 + AD Braak stages
Model 5: Model 4 + other co-pathologies (PD_Braak stages (0-II, III-IV, V-VI), CAA (absent/present), LBD (absent/present), hippocampal sclerosis (absent/present), TDP43(absent/present), infarcts (absent/present), arteriosclerosis (absent/present)

Abbreviations: N, number of individuals included in each model; CI, Confidence Interval; CDR-sob, Clinical Dementia Rating sum of boxes score; PD, Parkinson's Disease; LBD, Lewy Body Disease; TDP43, TAR DNA-binding protein 43; CAA, Cerebral Amyloid Angiopathy.

© 2026 Abu Raya M et al. *JAMA Neurology*.

eTable 9. Association of sex and amyloid pathology with cognitive abilities (CDR-SB) considering interactions between sex and amyloid (n=2,268)

Continuous Outcome: CDR-SB	Model 1		Model 2		Model 3		Model 4		Model 5	
	N=2268		N=1460		N=1443		N=1443		N=1443	
	β (95%CI)	p	β (95%CI)	p	β (95%CI)	p	β (95%CI)	p	β (95%CI)	p
**Sex**	0.15 (-0.58-0.88)	0.68	-0.20 (-0.91-0.51)	0.57	0.18 (-0.55-0.92)	0.63	0.17 (-0.54-0.87)	0.64	0.19 (-0.49-0.87)	0.59
**CERAD NP score (High A-LowA)**	4.03 (2.95-5.10)	**<.001**	2.81 (1.73-3.90)	**<.001**	2.66 (1.62-3.71)	**<.001**	0.88 (-0.17-1.94)	0.10	0.45 (-0.59-1.48)	0.40
**Sex* CERAD NP Score**	2.846 (1.44-4.25)	**<.001**	2.72 (1.36-4.08)	**<.001**	2.48 (1.16-3.80)	**<.001**	1.02 (-0.27-2.31)	0.12	1.13 (-0.12-2.38)	0.08
Abbreviations: N, number of individuals included in each model; CI, Confidence Interval; CDR-sb, Clinical Dementia Rating sum of boxes score; PD, Parkinson's Disease; LBD, Lewy Body Disease; TDP43, TAR DNA-binding protein 43; CAA, Cerebral Amyloid Angiopathy;										

Results from linear regression analyses assessing the association between sex and AD CERAD neuropathological scores and CDR-SB. Shown are the standardized estimates (β) for sex and CERAD neuropathological scores with 95% confidence intervals (CIs) and p-values. Male and 'None or Sparse' CERAD neuropathological scores are used as a reference. These estimates are from models that do not include the interaction term between sex and CERAD scores. Interaction effects are reported separately.

Models
Model 1: Outcome ~ sex + CERAD neuropathological score+ Sex* CERAD neuropathological score [(HighAβ-LowAβ)* (Female-Male)]
Model 2: Outcome ~ Model 1+age+race+education+ APOE4
Model 3: Outcome ~ Model 2 + cardiovascular risk factors
Model 4: Outcome ~ Model 3+ AD Braak stages
Model 5: Outcome ~ Model 4+ other co-pathologies (PD_Braak stages (0-II,III-IV,V-VI), CAA (absent/present), LBD (absent/present), hippocampal sclerosis (absent/present), TDP43(absent/present), infarcts (absent/present), arteriosclerosis (absent/present)

© 2026 Abu Raya M et al. *JAMA Neurology*.

eTable 10. Association of sex and amyloid pathology with cognitive abilities (CDR-Global) considering interactions between sex and amyloid

Results from ordinal logistic regression models examining the association between sex, amyloid pathology (CERAD neuropathological score), and global cognitive status measured by the Clinical Dementia Rating—Global score (CDR-Global). Shown are odds ratios (ORs) with 95% bias-corrected and accelerated (BCa) bootstrap confidence intervals (1,000 resamples) and p-values. Amyloid pathology was modeled as a binary variable: low Aβ (CERAD "None" or "Sparse") vs. high Aβ (CERAD "Moderate" or "Frequent"), with males and low amyloid as reference groups. Interaction effects between sex and amyloid pathology were tested in each model. Proportional odds assumptions were assessed using the test of parallel lines and were not violated across any model.

Ordinal Outcome: CDR-Global	Model 1		Model 2		Model 3		Model 4		Model 5	
	N=2268		N=1460		N=1443		N=1443		N=1443	
	OR (95%CI)	p	OR (95%CI)	p	OR (95%CI)	p	OR (95%CI)	p	OR (95%CI)	p
**Sex**	1.78 (1.47-2.21)	**<.001**	1.203 (0.94-1.58)	0.15	1.37 (1.03-1.85)	**0.03**	1.12 (0.86-1.58)	0.46	1.21 (0.86-1.95)	0.29
**AD CERAD NP score (High Aβ-LowAβ)**	5.40 (4.52-6.71)	**<.001**	3.91 (3.02-5.23)	**<.001**	3.86 (2.96-5.39)	**<.001**	1.75 (1.24-2.57)	**0.001**	2.02 (1.38-3.30)	**<.001**
**Sex* CERAD NP score**	1.68 (1.18-2.7)	**0.009**	1.83 (1.18-3.30)	**0.02**	1.77 (1.16-3.32)	**0.03**	1.22 (0.81-2.45)	0.47	1.38 (0.82-3.44)	0.34

Models:
Model 1: CDR-Global ~ sex + CERAD neuropathological score + sex × AD CERAD score
Model 2: Model 1 + age + race + education + APOE4
Model 3: Model 2 + cardiovascular risk factors
Model 4: Model 3 + AD Braak stage
Model 5: Model 4 + co-pathologies (PD Braak stage, CAA, LBD, hippocampal sclerosis, TDP-43, infarcts, arteriosclerosis)
Abbreviations:
CDR-Global, Clinical Dementia Rating Global score; CI, confidence interval; OR, odds ratio; CERAD, Consortium to Establish a Registry for Alzheimer's Disease; PD, Parkinson's disease; LBD, Lewy body disease; CAA, cerebral amyloid angiopathy; TDP-43, TAR DNA-binding protein 43

© 2026 Abu Raya M et al. *JAMA Neurology*.

eTable 11. Sensitivity analyses of the association between sex and amyloid deposition evaluated using the CERAD Neuropathological score across participants matched by functional cognitive level

	**Female Sex***									
	**Model 1**		**Model 2**		**Model 3**		**Model 4**		**Model 5**	
	**N**		**N**		**N**		**N**		**N**	
	**OR (95%CI)**	**p**	**OR (95%CI)**	**p**	**OR (95%CI)**	**p**	**OR (95%CI)**	**p**	**OR (95%CI)**	**p**
**AD CERAD score (0-3)** (Ordinal Outcome)										
**CDR-Global= 0**	1503		1503		994		985		**985**	
	1.55 (1.27-1.93)	**<.001**	1.22 (0.98- 1.60)	0.09	1.29 (1.01- 1.83)	0.09	1.32 (0.98- 1.87)	0.08	1.22 (0.89-1.73)	0.23
**CDR-Global= 0.5**	208		208		115		115		**115**	
	1.17 (0.73-2.03)	0.55	1.08 (0.67- 2.13)	0.78	1.74 (0.96- 4.77)	0.15	1.68 (0.81- 6.27)	0.20	1.26 (0.56-6.62)	0.60
**CDR-Global= 1**	119		119		64		64		**64**	
	0.92 (0.53-1.98)	0.81	0.92 (0.50- 2.24)	0.80	1.21 (0.61- 4.1)	0.68	Model did not converge		Model did not converge	
**CDR-Global= 2**	122		122		74		73		**73**	
	1.99 (1.16-4.96)	**0.06**	1.52 (0.81-4.75)	0.26	1.42 (0.70-6.08)	0.45	1.22 (0.58-21.21)	0.69	0.46 (0.14-0.744)	0.18
**CDR-Global= 3**	316		316		213		206		**206**	
	2.36 (1.59-4.16)	**<.001**	2.07 (1.40- 3.61)	**0.002**	2.48 (1.59- 5.02)	**0.002**	**2.78 (1.77- 7.68)**	**0.002**	**2.03 (1.12-10.07)**	**0.067**

Results from ordinal logistic regression analyses testing the association between sex and neuritic plaque burden (CERAD score: None → Sparse → Moderate → Frequent) among participants matched for cognitive status using CDR-Global scores. Odds ratios (ORs) for sex from the ordinal regression analysis are presented, with 95% confidence intervals (CIs) estimated from 1,000 bootstrap samples and corresponding P-values.

Models

Model 1: Unadjusted ordinal logistic regression model

Model 2: Ordinal logistic regression model adjusted for age, education, and reported race.

Model 3: Ordinal logistic regression model adjusted for age, education, race, and APOEε4.

Model 4: Ordinal logistic regression model adjusted for age, education, race, APOEε4, and † cardiovascular risk factors.

Model 5: Ordinal logistic regression model adjusted for age, education, race or AFR, APOEε4, †cardiovascular risk factors, and AD Braak stages.

*Male is used as the reference group

Abbreviations: CDR, Clinical Dementia Rating; N is the number of cases included in each model; APOE ε4, ε4 allele of *apolipoprotein E gene; CERAD score,* Consortium to Establish a Registry for Alzheimer's Disease

† Cardiovascular risk factors were defined as the presence (yes/no) of hypertension, diabetes, dyslipidemia, coronary artery disease, heart failure, stroke, or smoking. Body mass index (BMI) was included as a continuous variable.

Note: Sample sizes varied across cognitive subgroups; estimates for smaller strata (e.g., CDR 0.5–1) should be interpreted cautiously due to limited power.

© 2026 Abu Raya M et al. *JAMA Neurology*.

eReferences:

1. Instituto Brasileiro de Geografia e Estatística (IBGE). Cor ou Raça. Accessed May 2, 2024. https://educa.ibge.gov.br/jovens/conheca-o-brasil/populacao/18319-cor-ou-raca.html.

2. Travassos C, Williams DR. The concept and measurement of race and their relationship to public health: a review focused on Brazil and the United States. *Cad Saúde Pública*. 2004;20(3):660-678. doi:10.1590/S0102-311X2004000300003

3. Suemoto CK, Leite REP, Ferretti-Rebustini REL, et al. Neuropathological lesions in the very old: results from a large Brazilian autopsy study. *Brain Pathol Zurich Switz*. 2019;29(6):771-781. doi:10.1111/bpa.12719

4. Suemoto CK, Leite REP, Paes VR, et al. Neuropathological Lesions and Cognitive Abilities in Black and White Older Adults in Brazil. *JAMA Netw Open*. 2024;7(7):e2423377. doi:10.1001/jamanetworkopen.2024.23377

5. Suemoto CK, Ferretti-Rebustini REL, Rodriguez RD, et al. Neuropathological diagnoses and clinical correlates in older adults in Brazil: A cross-sectional study. Brayne C, ed. *PLOS Med*. 2017;14(3):e1002267. doi:10.1371/journal.pmed.1002267

6. Braak H, Tredici KD, Rüb U, De Vos RAI, Jansen Steur ENH, Braak E. Staging of brain pathology related to sporadic Parkinson's disease. *Neurobiol Aging*. 2003;24(2):197-211. doi:10.1016/S0197-4580(02)00065-9

7. Grinberg LT, Thal DR. Vascular pathology in the aged human brain. *Acta Neuropathol (Berl)*. 2010;119(3):277-290. doi:10.1007/s00401-010-0652-7

8. Calero O, Hortigüela R, Bullido MJ, Calero M. Apolipoprotein E genotyping method by Real Time PCR, a fast and cost-effective alternative to the TaqMan® and FRET assays. *J Neurosci Methods*. 2009;183(2):238-240. doi:10.1016/j.jneumeth.2009.06.033

9. Falush D, Stephens M, Pritchard JK. Inference of population structure using multilocus genotype data: dominant markers and null alleles. *Mol Ecol Notes*. 2007;7(4):574-578. doi:10.1111/j.1471-8286.2007.01758.x

10. Schlesinger D, Grinberg LT, Alba JG, et al. African ancestry protects against Alzheimer's disease-related neuropathology. *Mol Psychiatry*. 2013;18(1):79-85. doi:10.1038/mp.2011.136

11. Naslavsky MS, Suemoto CK, Brito LA, et al. Global and local ancestry modulate APOE association with Alzheimer's neuropathology and cognitive outcomes in an admixed sample. *Mol Psychiatry*. 2022;27(11):4800-4808. doi:10.1038/s41380-022-01729-x

12. The jamovi project (2022). jamovi. (Version 2.3) [Computer Software]. Retrieved from https://www.jamovi.org.

13. Hunter JD. Matplotlib: A 2D Graphics Environment. Comput Sci Eng 2007;9:90–5. https://doi.org/10.1109/MCSE.2007.55.

© 2026 Abu Raya M et al. *JAMA Neurology*.
