# Supplementary material for: Sex Differences in Amyloid Pathology by Race, Ancestry, and Apolipoprotein E ε4 in an Admixed Autopsy Sample
Source: JAMA Neurol. 2026 Feb 23;83(4):392–401. doi: 10.1001/jamaneurol.2026.0054 (PMC12931469; doi:10.1001/jamaneurol.2026.0054)
Supplement: Supplement 2. — Data sharing statement [file jamaneurol-e260054-s002.pdf]

Data Sharing Statement

Abu Raya. Sex Differences in Amyloid Pathology by Race, Ancestry, and Apolipoprotein E ε4 in an Admixed Autopsy Sample. *JAMA Neurol*. Published February 23, 2026.
doi:10.1001/jamaneurol.2026.0054

Data
Data available: Yes
Data types: Deidentified participant data, Data dictionary
How to access data: data will be shared upon request to the corresponding author - grinberg.lea@mayo.edu
When available: With publication

Supporting Documents
Document types: Statistical/analytic code
How to access documents: data will be shared upon request to the corresponding author - grinberg.lea@mayo.edu
When available: With publication

Additional Information
Who can access the data: researchers whose proposed use of the data has been approved
Types of analyses: specified purpose
Mechanisms of data availability: after approval of a proposal, and with a signed data access agreement)
